# Toward the Existence of a Sympathetic Neuroplasticity Adaptive Mechanism Influencing the Immune Response. A Hypothetical View—Part II

**DOI:** 10.3389/fendo.2019.00633

**Published:** 2019-09-18

**Authors:** Emanuel Bottasso

**Affiliations:** Departments of Pathology and Physiology, Faculty of Medicine, Centro de Altos Estudios en Ciencias Humanas y de la Salud, Universidad Abierta Interamericana, Rosario, Argentina

**Keywords:** neuro-immune interaction, sympathetic nervous system, inflammation, neural plasticity, peripheral immune tolerance

## Abstract

In the preceding work, a hypothesis on the existence of a specific neural plasticity program from sympathetic fibers innervating secondary lymphoid organs was introduced. This proposed adaptive mechanism would involve segmental retraction and degeneration of noradrenergic terminals during the immune system (IS) activation followed by regeneration once the IS returns to the steady-state. Starting from such view, this second part presents clinical and experimental evidence allowing to envision that this sympathetic neural plasticity mechanism is also operative on inflamed non-lymphoid peripheral tissues. Importantly, the sympathetic nervous system regulates most of the physiological bodily functions, ranging from cardiovascular, respiratory and gastro-intestinal functions to endocrine and metabolic ones, among others. Thus, it seems sensible to think that compensatory programs should be put into place during inflammation in non-lymphoid tissues as well, to avoid the possible detrimental consequences of a sympathetic blockade. Nevertheless, in many pathological scenarios like severe sepsis, chronic inflammatory diseases, or maladaptive immune responses, such compensatory programs against noradrenergic transmission impairment would fail to develop. This would lead to a manifest sympathetic dysfunction in the above-mentioned settings, partly accounting for their underlying pathophysiological basis; which is also discussed. The physiological/teleological significance for the whole neural plasticity process is postulated, as well.

## Introduction

In the preceding work (1) evidence regarding changes in the sympathetic innervation of secondary lymphoid organs (SLOs) during the activation of the immune system (IS) was presented. Different authors interpreted this phenomenon as “damage” or “injury” of the noradrenergic axons, probably due to the action of endogenous mediators. In contrast to this view, the hypothesis of a neural plasticity adaptive mechanism was postulated –involving axonal degeneration during the activation of the IS with subsequent axonal regeneration once the immune response ceases, thus recovering the innervation pattern of the steady-state. It was also proposed that this mechanism may be mediated by molecules such as neurotrophins and semaphorins.

One of the main remaining questions was whether these changes in innervation would also occur in other non-lymphoid organs and tissues during inflammation, encompassing recruitment of immune cells and/or presence of inflammatory cytokines. Given that the above-mentioned molecules mediating neural plasticity can be produced by immune cells or by other different cell types under cytokine influence (2–6), this hypothetical view seems plausible. In support of this, clinical and experimental evidence regarding the loss of sympathetic innervation during different inflammatory conditions is now presented. As part of the autonomic nervous system (ANS), the sympathetic nervous system (SNS), regulates nearly all bodily functions (7). Hence, a sympathetic dysfunction would become clinically manifest, in cases wherein a compensation against this hypothetical impaired noradrenergic transmission is insufficient, implying life-threatening consequences in some circumstances.

Nature is unlikely to orchestrate complex and energy-wasting mechanisms for nothing. As the nervous system (NS) regulates most phases of the immune response, mainly through the SNS (8–11), the immunological meaning for this postulated retraction of the noradrenergic terminals both in SLOs and in non-lymphoid tissues during immune-mediated processes is also proposed.

### Sepsis and Septic Shock

Sepsis is one of the main causes of morbidity and mortality throughout the world consisting of a dysregulated systemic inflammatory response syndrome against a specific pathogen infection. Sepsis with organic dysfunction is called severe sepsis, which can progress to septic shock, characterized by persistent hypotension <65 mmHg leading to a state of acute circulatory failure (12–15). Organic dysfunction in severe sepsis can include renal, hepatic, cardiac or pulmonary failure, lactic acidosis, thrombocytopenia with abnormalities in coagulation or multiple organ failure. Bacterial endotoxins such as LPS activate the NF-κB pathway in immune cells with the subsequent production and release to the circulation of inflammatory mediators such as TNF, IL-1, IL-6, IL-8, and macrophage migration inhibitory factor, presumably involved in the above-referred clinical alterations.

Vasoplegia and myocardial dysfunction are the two complications of septic shock leading to hemodynamic instability (16, 17). Vasoplegia is defined as a lack of vasculature response to vasopressors (18, 19) leading to a state of persistent peripheral vasodilation, hypotension, and hypoperfusion. Nitric oxide (NO), synthesized by the vascular smooth muscle inducible nitric oxide synthase (iNOS) under the control of cytokines, may play a central role in this regard (20). As to cardiac function, at the beginning of sepsis, patients have a hyperdynamic phase characterized by an increased cardiac out-put as a reaction to the decreased peripheral vascular resistance. After that, progression toward septic shock is characterized by a depressed activity of the ventricular myocardium along with a reduced ejection fraction. Since this depression cannot be simply explained by hypoperfusion and coronary ischemia, a direct action of inflammatory mediators, as depressants, was postulated (21–23). It is currently believed that such depression is multifactorial, involving metabolic alterations and mitochondrial dysfunction of the cardiomyocytes, reduced calcium release from the sarcoplasmic reticulum and impaired electromechanical coupling at the myofibrillar level (17). These alterations seem to be caused by different cytokines produced and released from activated immune cells, as well as NO.

The first-line treatment for the maintenance of hemodynamic stability in septic shock is norepinephrine (NA) -or other sympathomimetics such as dopamine or dobutamine through their effects on α- and β-adrenoreceptors (ARs) and their high vasoconstrictive action and inotropic effect on the vascular and cardiac muscle, respectively (12–15). Vasopressin can also be used, to reduce NA doses.

**Figure 1:**
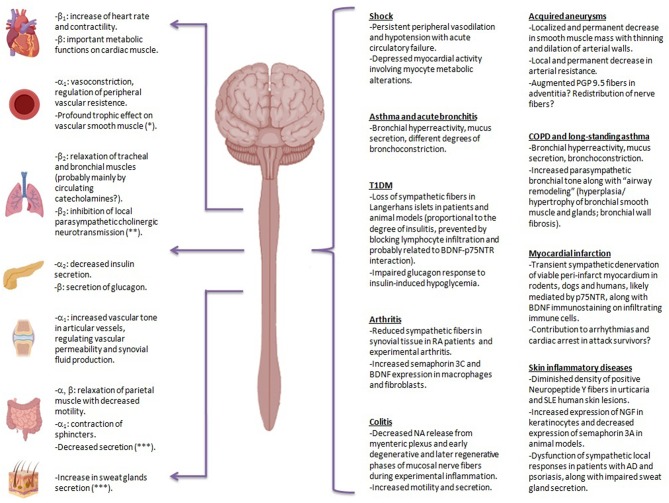
Clinical and experimental evidence of sympathetic structural and functional alterations in many inflammatory scenarios. In different situations in which compensatory mechanisms against sympathetic blockade may be insufficient or may have failed to evolve, sympathetic dysfunction is likely to become evident. This may apply to chronic inflammatory diseases, the ones due to hypersensitivity reactions (maladaptive immune reactions *per se*), and situations of protracted and dysregulated immune responses failing to eradicate pathogens, i.e., prolonged septicemia. Physiological effects of sympathetic nervous system on different organs are depicted on the left, together with the involved adrenergic-receptor. Evidence suggestive of sympathetic impairment in different pathological conditions is shown on the right. PGP 9.5, protein gene product 9.5; COPD, chronic obstructive pulmonary disease; T1DM, type 1 diabetes mellitus; BDNF, brain-derived neurotrophin factor; p75NTR, p75 neurotrophin receptor; RA, rheumatoid arthritis; SLE, systemic lupus erythematosus; NGF, nerve growth factor; AD, atopic dermatitis: NA, norepinephrine. *Mediated by sympathetic adrenergic and non-adrenergic transmission.**Parasympathetic cholinergic neurotransmission elicits in turn bronchoconstriction, increases mucus production and favors airway remodeling (through muscarinic mediated proliferation of bronchial smooth myocytes and fibroblasts). ***Mediated by sympathetic non-adrenergic transmission.

Sympathetic noradrenergic fibers normally mediate vasoconstriction by acting on α_1_-ARs from the smooth muscle of arteries and veins, thus regulating peripheral vascular resistance. On the other hand, by acting on β_1_-ARs, the sympathetic activity increases myocardial contractility -both atrial and ventricular- as well as the heart rate (7). As commented, during sepsis and septic shock inflammatory mediators can lead to vasodilation and a decrease in peripheral vascular resistance as well as depression of myocardial activity. Regardless of the action of inflammatory mediators, the question emerges as to why the SNS fails to overcome this alteration to maintain hemodynamic stability, raising the need for exogenous sympathomimetics administration to keep the patient alive. It follows that some impairment in the noradrenergic transmission is likely to exist during sepsis and septic shock. Considering that sepsis is a systemic inflammatory response, it may be hypothesized that even in the absence of immune cells infiltrating the vessel walls or the heart, circulating inflammatory mediators favor a probable retraction of the noradrenergic terminals, leading to an impairment in sympathetic transmission, as it may happen in SLOs during IS activation (1). In line with the proposed hypothesis, this impairment may be due to the action of neurotrophins and semaphorins with possible re-expression of p75 neurotrophin receptor (p75NTR) in vascular and cardiac sympathetic nerves in an inflammatory milieu (Figure 1). These molecules might be locally produced under the influence of cytokines, as found in different tissues (2–6). A possible action of netrin-1, an axon guidance molecule able to mediate neural fibers retraction, expressed in epithelial and endothelial cells under inflammatory influences, may not be discarded (24, 25); in addition to a probably direct action of some inflammatory cytokines, given their regulatory role in neurogenesis and synaptic function (26, 27).

This sympathetic dysfunction may not only explain the lack of vasoconstriction reflex, but also other alterations observed in sepsis and septic shock, like myocardial metabolic alterations and reduced intracellular calcium in cardiomyocytes, contributing to a decreased contractility (Figure 1). In fact, sympathetic action has very important metabolic functions since NA increases glucose uptake in brown fat and heart (28, 29), by mechanisms other than insulin (30), like the important enhancement of GLUT1 functional activity (31). NA also stimulates glucose utilization by myocytes (32), thyrocytes and platelets. As to calcium levels, endotoxins and cytokines alter and suppress L-type calcium currents in cardiomyocytes, possibly via changes in the autonomic regulation of this channel (33–35). Calcium trafficking is also linked to mitochondrial function and integrity (17).

Different authors raised the view of an autonomic dysfunction in multiple organ failure as contributing significantly to the pathogenesis of this syndrome (36–38). In fact, a decreased sympathetic activity has been observed in the early course of severe sepsis that may contribute to circulatory and cardiac failure (39). In anesthetized cats, the injection of *Escherichia coli* endotoxin causes a significant decrease in mean blood pressure with a drop in sympathetic activity of the splanchnic nerve (40). Post-mortem examinations in humans dying from septic shock reveal neuronal and glial apoptosis within cardiovascular autonomic centers with a significantly increased brain expression of TNF and iNOS (41). Provided a noradrenergic transmission blockade does occur in sepsis, as part of the proposed adaptive neural plasticity mechanism involving sympathetic decreased activity during inflammation, the question remains on how it has evolved when causing hemodynamic instability and even patient death? Being so, other compensatory mechanisms must also exist tending to maintain peripheral vascular resistance and cardiac functionality. Progression toward septic shock with hemodynamic instability may then represent the exhaustion of compensatory programs given the immune incapability to eliminate the pathogen and the persistence of systemic inflammation.

Activation of the renin-angiotensin-aldosterone system is a well-characterized physiologic mechanism to prevent hypotension during sepsis (42). Also, it is worth reminding that vertebrates have two main sources of NA and adrenaline (Adr): the sympathetic nerves and the adrenal medulla. The function of the catecholamines released by the adrenal medulla in the septic scenario is yet poorly understood. Perhaps it may be basically compensatory to preserve hemodynamic stability given the transient blockade of noradrenergic transmission, mainly at the vascular level. Thus, the perpetuation of the sympathetic blockade may exhaust the adrenal medulla in its compensatory attempt along with a certain degree of tissue hypo-responsiveness due to prolonged exposure to circulating catecholamines favoring circulatory instability. In parallel, immunoinflammatory responses are known to also activate the hypothalamic-pituitary-adrenal (HPA) axis, leading to the release of corticosteroids, a major immunomodulatory compound (43, 44). Adrenal insufficiency, at least in terms of corticosteroid production, is present during sepsis (45), and likely detrimental in this regard considering that corticosteroids regulate vascular reactivity to vasoconstrictors (46).

The adrenal medulla releases both NA and Adr, in a very variable proportion depending on species [for review, (47)]. There are also two independent sympathetic innervation pathways in the adrenal medulla, one mediating the release of Adr and the other one NA. Rat preganglionic sympathetic neurons innervating the Adr-releasing adrenal cells are not influenced by baroreceptor arterial reflexes but stimulated by hypoglycemia. Unlike this, preganglionic neurons that innervate the noradrenergic cells from the adrenal medulla are under a potent baroreceptor arterial reflex control. Thus, Adr released from the adrenal medulla to circulation primarily exerts metabolic effects by mediating glycogenolysis in the liver and skeletal muscle, with no significant effect on the maintenance of circulatory homeostasis. On the other hand, NA released into the plasma from the adrenal medulla has neither metabolic nor hemodynamic effects under physiological conditions. In this sense, it is important to note that, in neuro-effector vascular junctions, NA reaches concentrations in the micromolar range, while circulating NA barely reaches concentrations in the picomolar range, being normally unable to exert any effect. However, under pathophysiological circumstances, this situation changes markedly. In fact, after sympathetic denervation, some effector organs, like vascular smooth and cardiac muscles, develop an adaptive hyper-responsiveness to adrenal circulating NA, viewed as a compensatory mechanism (48).

During sepsis and septic shock, both Adr and NA plasma levels and ARs expression in different tissues undergo important and varied modifications. In this sense, high levels of circulating catecholamines have been observed during human and experimental sepsis (39, 49–51). On the other hand, the α_1_-ARs from human hepatocytes experience dynamic changes during sepsis showing an increased, normal or decreased expression in mild, moderate, or severe sepsis, respectively (52). In septic rats, myocardial ARs were found to be decreased (53, 54). From a hypothetical viewpoint, these variations throughout the sepsis spectrum may reflect an initial blockade of sympathetic neurotransmission and a subsequent over-exposure to circulating adrenal catecholamines.

### Anaphylaxis and Anaphylactic Shock

The term anaphylaxis is used to describe a rapid and widespread immunological reaction occurring after exposure to certain substances in previously sensitized persons [for review (55, 56)]. The most frequent triggers are food, medicines, and insect bites, causing a type I immediate hypersensitivity reaction. Clinically, the most common life-threatening manifestations include angioedema, pulmonary edema, bronchospasm, and hemodynamic instability in cases of anaphylactic shock, characterized by hypotension due to decreased peripheral vascular resistance, and occasionally myocardial depression.

Cells implicated in this reaction are mast cells and granulocytes, which upon degranulation release pre-formed and newly and rapidly synthesized inflammatory mediators into the systemic circulation. Among these mediators, the most important ones are histamine, tryptase, chymase, bradykinin, and heparin as well as metabolites from the arachidonic acid, including products of the lipoxygenase and cyclooxygenase pathways such as prostaglandins and leukotrienes. During severe anaphylaxis episodes, there is concomitant activation of complement and coagulation pathways, and the kallikrein-kinin contact system. These mediators are capable of inducing vasodilation and mucosal edema, bronchial smooth muscle contraction, and increased mucus production. As in septic shock, the first-line treatment is the administration of fluids and sympathomimetics like Adr (57, 58), which reverses all features of anaphylaxis. In fact, stimulation of α-ARs increases peripheral vascular resistance, raising the blood pressure while reversing peripheral vasodilation and decreasing angioedema. Stimulation of β_1_-ARs has positive inotropic and chronotropic effects on the cardiac muscle, whereas β_2_-ARs stimulation leads to bronchodilation. β-ARs also increase the production of intracellular cyclic AMP, which stabilizes mast cells, inhibiting further mediator release. At this point, one might wonder why the organism does not respond with an increased sympathetic tone to such a massive systemic release of these inflammatory mediators and hence maintaining homeostasis with no need of exogenous catecholamine administration. Once again, there may exist a blockade of sympathetic transmission during anaphylactic shock, probably mediated by mechanisms like those proposed for septic shock (Figure 1). Several years ago, the group of Levi-Montalcini described the production, storage, and release of nerve growth factor (NGF) by mast cells, suggesting interactions between the NS and IS (59). Probably the adrenal production and release of catecholamines may be insufficient in these cases to counterbalance the SNS dysfunction in front to such a massive maladaptive reaction.

Supporting the hypothesis of the sympathetic transmission blockade in the pathophysiology of distributive shock, it is worth remembering that the disruption of descending pathways from central centers to spinal sympathetic neurons may also lead to hemodynamic instability. In fact, spinal cord injury provokes different clinical manifestations that will depend on the localization and severity of the lesion, with some patients developing neurogenic shock in the acute phase, and even multi-organ dysfunction syndrome, mainly in severe cervical lesions (60). The initial response in these cases consists of a massive sympathetic stimulation and parasympathetic reflex activity lasting 3–4 min due to the release of catecholamines by adrenal glands immediately after the injury. This results in severe hypertension and heart rhythm alterations. After this short initial phase, there is a massive decrease in sympathetic activity, with a reduction in peripheral vascular resistance, marked hypotension and, occasionally, bradycardia due to the absence of sympathetic tone and unimpeded vagal tone, which characterizes the state of shock (61, 62). In contrast, during the chronic phase, some patients display autonomic dysreflexia, after spinal shock resolution, which constitutes a life-threatening syndrome of massive imbalanced reflex sympathetic discharge.

It follows that the solely traumatic lesion of the sympathetic pathway reproduces, in the acute phase, a hemodynamic instability state, like the one seen in sepsis or anaphylaxis (Figure 1). Furthermore, the treatment of neurogenic shock is also based on the replacement of volume and the administration of sympathomimetic vasopressors.

### Acquired Vascular Aneurysms

One of the most frequent forms of acquired aneurysms is the abdominal aortic aneurysm—AAA—(63). AAA consists of a localized and permanent weakening and dilation of this vessel over 50% of its usual diameter or >3 cm, which in most cases compromises the infrarenal segment. In 65–80-year old men, the prevalence is between 1–2 and 8% according to the series; being 6 times less frequent in women [for review (64, 65)]. The most important complication is the rupture, which leads to significant bleeding and an estimated 150,000–200,000 deaths yearly worldwide.

Although the pathogenesis of AAA is not yet clearly elucidated, most researchers agree that its development is related to atherosclerosis along with chronic inflammation (63). In fact, atherosclerosis, previously considered as a disease of lipid storage, involves an important inflammatory response, with presence within the arterial wall of cells from innate and adaptive immunity, and locally produced cytokines (66–69). Moreover, targeting of inflammatory adhesion molecules reduces atherosclerosis, whereas, removing or blocking IL-10 or TGF-β accelerates its development.

Apparently, the release of proteolytic enzymes, oxidation-derived free radicals and cytokines during such a chronic inflammatory response leads to a reduction in elastin content, a distorted elastin configuration, increased deposition of type I collagen and reduced type III collagen, in both tunica media and adventitia. These phenomena may also diminish the number of smooth muscle cells leading to a marked thinning of the tunica media, typical of AAA, resulting in a decreased resistance of the arterial wall (70, 71). Search into the literature revealed one study on the AAA innervation through immunohistochemistry for protein gene product 9.5 (PGP 9.5), indicating an apparent increase in the number of nerve fibers in AAA only in the adventitia (71), without identifying the proper fiber type. Beyond this fact, the question remains whether this increased immunostaining does correspond to a real increase in nerve terminals or to a redistribution within the AAA wall.

Not only in AAA but also in other pathological settings, i.e., tertiary syphilis and “mycotic” aneurysms, the existence of localized chronic inflammation of the arterial wall results in thinning of the tunica media and dilation of the vessel with aneurysm formation. Noticeably, the occurrence of these pathologies is very low nowadays compared to the one recorded in the pre-antibiotic era (72–75).

As it is widely known, the SNS innervates the vascular smooth muscle leading to vasoconstriction by acting on α_1_-ARs, thus increasing flow resistance in large and small arteries and arterioles (7). Beyond this effect, *in vivo*, and *in vitro* studies showed that sympathetic fibers exert a profound trophic effect on vascular smooth muscle, stimulating its proliferation and differentiation, probably not only through NA but also through co-transmitters such as ATP and neuropeptide Y (76–81).

Within the setting of the proposed adaptive neural plasticity mechanism, chronic inflammation of the vessel wall may mediate a retraction of the sympathetic fibers and probably the apoptosis of neurons innervating such arterial segment, through p75NTR stimulation (82–84). Considering the localized and chronic nature of the inflammatory process, sympathetic denervation may lead to a marked decrease in the trophism of the tunica media of the arterial wall, which may partly explain the weakening and dilation of vessels, with the ensuing aneurysm formation (Figure 1).

### Asthma

Asthma is a heterogeneous inflammatory disease of the lower airways causing recurrent symptoms and exacerbations. It can develop at any age, but the disease onset is more frequent in childhood or young adulthood, affecting about 7.5% of the adult population [reviewed in (85–87)]. It is characterized by bronchial hyperreactivity, cough, mucus secretion, different degrees of bronchoconstriction and dyspnea. Even when allergic and non-allergic phenotypes are described (88, 89), the immunopathological characteristics of both patient groups are similar and this distinction is not easy. The bronchial mucosa is infiltrated by a series of inflammatory cells, like eosinophils, mast cells, neutrophils, and lymphocytes.

The chemical mediators released by the inflammatory cells in the context of an immediate hypersensitivity reaction (like histamine and arachidonic acid metabolites) seem to be the cause of asthma symptoms. Nevertheless, it has long been thought that the mechanisms put into place by such mediators, linked to disease symptoms, may be of neural nature. For instance, the β-adrenergic blocking theory proposed by Szentivanyi (90) argued that a diminished responsiveness to β-adrenergic stimulation could increase impulse transmission or receptor stimulation through α-adrenergic or cholinergic pathways. Since then an important body of evidence suggests the existence of an autonomic dysfunction in patients with asthma. Some groups have observed increased bronchial cholinergic responsiveness and β-adrenergic hyporesponsiveness (91), which would lead to bronchospasm, mucosal edema, augmented mucous secretion, cough, and dyspnea (92). Moreover, according to some authors, not only asthma symptoms but also the most common ones seen in other respiratory diseases may be explained by a dysfunction in the ANS (93, 94). Furthermore, asthma treatment is currently addressed to reduce inflammation through local or systemic corticosteroids as well as to promote β_2_-adrenergic stimulation or cholinergic inhibition. Other drugs like leukotriene receptor antagonist and leukotriene synthesis inhibitor, along with biological therapies such as antibodies against IgE or IL-5 are also employed (86, 87).

The autonomic innervation of the lower airway is somewhat complex, regulating tones from the bronchial smooth muscle, the vessel wall and the activity of bronchial glands (7). Parasympathetic nerves are the dominant neural pathway in the control of airway smooth muscle tone and mucus secretion in humans, with acetylcholine (ACh) acting on type 3 muscarinic receptors, promoting bronchoconstriction (95). The sympathetic innervation in the human airways is fundamentally present in the vicinity of the submucosal glands and the bronchial arteries. The bronchial smooth muscle, on the other hand, does not appear to be directly innervated by adrenergic fibers. However, β-ARs, which mediate bronchorelaxation, are widely distributed in the human lung. It has been postulated that circulating Adr may act on these receptors facilitating the dilation of the bronchial smooth muscle, but there is no convincing evidence in this regard. Nevertheless, the SNS does influence bronchial muscle tone through adrenergic fibers indirectly (96). In fact, adrenergic transmission can inhibit cholinergic neurotransmission at different levels. In parasympathetic ganglia, which are predominantly and physically associated with larger airways, sympathetic nerves stimulate ARs, thus preventing cholinergic activity (97). Moreover, in the bronchial walls themselves, sympathetic fibers end on parasympathetic postganglionic nerves, probably inhibiting cholinergic output through stimulation of prejunctional β_2_-ARs (98). In this sense, it is well-known that the sympathetic blockade induced by treatment with β-blockers produces bronchospasm and precipitates asthma (99, 100). This effect is thought to be caused by blockade of presynaptic β_2_-ARs on cholinergic nerves, which normally inhibits ACh release (98). In addition to the cholinergic and adrenergic fibers, a non-adrenergic non-cholinergic nervous system exists in the airways, exhibiting inhibitory (bronchodilator) or excitatory (bronchoconstrictor) actions (95).

To support the hypothesis that symptoms of inflammatory airway diseases are caused by an autonomic dysfunction, some authors have postulated that the different mediators produced and released locally during an inflammatory response may stimulate action potential discharge in parasympathetic nerves leading to bronchoconstriction (93). Even if this turns out to be true, it cannot be excluded that a primary decreased adrenergic transmission leads to increased cholinergic activity, thus contributing to the development of asthma, within the hypothetical mechanism involving neurotrophins and semaphorins effects on sympathetic nerves (Figure 1). As commented, these molecules can be produced by the immune cells themselves or by bronchial smooth muscle cells under cytokine influence (2). As proposed for anaphylaxis, compensatory programs against a possible noradrenergic transmission blockade may simply be insufficient to counterbalance bronchoconstriction in asthma, due to the maladaptive type of the immune response, predominantly immediate hypersensitivity.

### Bronchial Hyperreactivity During Acute Inflammation of the Airways

Formerly healthy subjects undergoing viral infections in the respiratory tract are largely known to experience bronchial hyperreactivity without developing clinical asthma. In fact, the inhalation of histamine diphosphate aerosol produces a significantly higher increase in airway resistance from normal subjects with flu, compared to the increase recorded in healthy individuals. Moreover, isoproterenol hydrochloride (a β-adrenergic agonist), and atropine sulfate aerosol (a muscarinic antagonist) inhibit and reverse such increased histamine-induced airway resistance, implying that increased cholinergic and/or decreased adrenergic activity play a role in the contraction of the smooth bronchial muscle in this scenario (101, 102).

In parallel, it is well-known that bronchoconstriction and wheezing resulting from increased bronchial reactivity are much more frequent in childhood during viral respiratory diseases caused by the respiratory syncytial virus (RSV), human metapneumovirus, rhinovirus, parainfluenza virus, influenza virus, and adenovirus, among others (103). Also, RSV infection of the lower respiratory tract in children is associated with an increased risk for the subsequent development of recurrent asthma/wheezing, becoming less likely as age increases (104). Such association was seen in viral respiratory infections other than the one caused by RSV, as well (105).

The reason for this persistence of bronchial hyperreactivity beyond the resolution of the infectious disease in early childhood is not currently understood. Viral infections may increase asthma susceptibility by acting onto the neural control of the respiratory tract. Indeed, the airway inflammation may lead to some degree of blockade of adrenergic transmission, thus facilitating cholinergic transmission, through the proposed neural plasticity mechanism (Figure 1). In theory, previously healthy adults would not experience bronchial obstruction, but rather a certain degree of bronchial hyperreactivity, probably due to the existence of compensatory programs. By opposite, symptoms may be more florid in early childhood, when the NS is still developing. Moreover, since the p75NTR is capable to mediate neuronal apoptosis (82–84), it may be speculated that severe airway inflammation in early developmental stages alters the normal innervation of the respiratory tract, perhaps further progressing to wheezing or asthma, as described in children.

### Chronic Obstructive Pulmonary Disease (COPD) and Long-Standing Asthma

COPD comprises a heterogeneous group of pathologies affecting the respiratory tract and pulmonary parenchyma, such as chronic bronchitis and emphysema, which are characterized by an incompletely reversible obstruction to the expiratory flow [for review (106–108)]. Although there are many risk factors, most cases are smoking-related and develop after the fourth decade of life. It manifests with periodic exacerbations due to viral or bacterial respiratory infections, causing an estimated of 3.2 million people deaths yearly worldwide.

Although in asthma airflow obstruction is usually intermittent and reversible, it can progress to an irreversible obstructive pattern, like COPD, in older people with a history of long-standing asthma. Thus, asthma and COPD overlap and converge, sharing three basic common clinical features: airway inflammation and obstruction along with bronchial hyperresponsiveness (109–112). Like asthma, COPD treatment is based on the use of inhaled corticosteroids and bronchodilators such as β-adrenergic and anticholinergic drugs, as well as oxygen therapy in some cases (106).

Regardless of the type of immune response seen in COPD or asthma, airway chronic inflammation may lead by itself to bronchial histo-structural changes usually referred to as “airway remodeling” partly accounting for the phenotypic clinical overlap of asthmatic and COPD patients (109, 113). Although there exist some differences in remodeling patterns between COPD and asthma, both cases are characterized by increased thickness of the basal membrane, changes in the extracellular matrix (fibrosis of the bronchial wall), angiogenesis, increased permeability of mucosal vessels, with hyperplasia/hypertrophy of glandular structures and the bronchial smooth muscle. This increased airway smooth muscle mass is the most important contributor to airway hyperresponsiveness and obstruction (114).

As well as the parasympathetic bronchial tone was found increased in patients with COPD and asthma, non-neuronal cells -including inflammatory cells and airway structural cells- can synthesize and release ACh (115, 116). Beyond its traditional role as a bronchoconstrictor, ACh may also play a pro-inflammatory immunological role favoring airway remodeling via pro-fibrotic and pro-proliferative mechanisms mediated by muscarinic receptors. In fact, stimulation of muscarinic receptors induces the proliferation of fibroblasts and collagen production. Notably, ACh may play an important role in the increase of bronchial smooth muscle mass, by enhancing the effect of different mediators (TGF-β, epidermal growth factor and platelet-derived growth factor) on proliferation, hypertrophy, and differentiation of smooth myocytes of the airway wall. Current clinical and experimental evidence suggests that anticholinergic drugs, mostly the long-acting tiotropium bromide, may reduce airway remodeling and the degradation of lung function, beyond its bronchodilator effect (117, 118).

As proposed for asthma and acute respiratory infections, chronic bronchial inflammation may lead to a blockade of sympathetic adrenergic transmission, giving rise to a sustained and unopposed increased parasympathetic cholinergic tone, responsible for the irreversibility of the bronchial hyperreactivity, mucus production and bronchoconstriction observed in COPD and long-standing asthma (Figure 1). The chronic nature of this condition would have prevented any adaptive compensatory program from evolving. Furthermore, since p75NTR can mediate neuronal apoptosis (82–84), the adrenergic innervation may be definitively lost in this context.

### Type 1 Diabetes Mellitus

Diabetes mellitus is characterized by a deregulation of carbohydrate, lipid and protein metabolism. There are two major types of diabetes, type 1 (T1DM) and type 2 (T2DM). T2DM is the most common form (accounting for more than 90% of cases) and develops in adult life over a background of peripheral insulin resistance with subsequent exhaustion of pancreatic β cells to produce it (119). On the other hand, T1DM is an autoimmune disease (120), presumably caused by T cell-mediated destruction of pancreatic β cells, with inflammation of the islets of Langerhans—insulitis—composed of T cells, B cells, macrophages and dendritic cells—DCs—(121–124).

The ANS innervates the islets of Langerhans, contributing to the regulation of endocrine pancreas function (7, 125). Accordingly, parasympathetic nerves stimulate insulin secretion whereas sympathetic nerves inhibit basal and glucose-stimulated insulin secretion. Regarding autonomic regulation of glucagon secretion, there are three mechanisms of direct stimulation of adrenergic and cholinergic receptors from α cells, which are activated in the brain during hypoglycemia and tend to facilitate glucagon release to normalize glycemia: (1) sympathoadrenal system, which culminates with the release of Adr by the adrenal medulla; (2) islet parasympathetic nerves, eliciting a modest glucagon response; (3) islet sympathetic nerves, that provoke a robust glucagon response (126, 127). It is known that the physiological glucagon response to insulin-induced hypoglycemia is impaired in T1DM, but not in T2DM (128). Interestingly, Taborsky's group linked this impaired glucagon response to a loss of sympathetic innervation of Langerhans islets in the context of the T1DM insulitis (129).

In fact, rodent models of T1DM (Bio-Breeder rats and NOD mice), in which an impaired glucagon response to insulin-induced hypoglycemia is also observed, showed an early and selective loss of sympathetic innervation in the islets of Langerhans. Such loss does not affect the exocrine pancreas and has not been observed in other models of non-autoimmune diabetes, like streptozotocin-induced diabetes (130, 131). In the above-referred T1DM models, the loss of sympathetic fibers is fully established in the first 2–3 weeks after the onset of diabetes and does not progress any further, thus differing from diabetic neuropathy, due to chronic hyperglycemia, which develops much later in the course of this disease and does progress. It was also shown that islet sympathetic nerve loss was proportional to the degree of invasive insulitis and that could be prevented by blocking lymphocyte infiltration. In another experimental animal model, they demonstrated that p75NTR was required for the loss of islet sympathetic fibers during insulitis, since mice lacking p75NTR retained most of their islet sympathetic nerves (132). Further studies in pancreas necropsy samples from patients with T1DM and T2DM and non-diabetic controls, revealed a severe loss of sympathetic fibers in islets of T1DM patients, either of recent onset (<2 weeks) or long-term disease (>10 years). This loss was observed neither in patients with T2DM nor in the exocrine pancreas of both patients with T1DM and T2DM (133). Apparently, such early loss of islet sympathetic nerves may be mediated by the brain-derived neurotrophic factor (BDNF) acting on p75NTR, being unclear which cells within the infiltrated islets of Langerhans will produce this neurotrophin (134).

As proposed in other cases of chronic inflammation, it may be hypothesized that semaphorins and pro-neurotrophins, produced by immune cells infiltrating the islets of Langerhans in T1DM–or by other cytokine-influenced local cells–act on sympathetic nerves, thus mediating their retraction. P75NTR stimulation may also induce neural apoptosis, with a definitive loss of sympathetic islet innervation (82–84). According to Taborsky's group (129), this loss of sympathetic innervation may partly explain the impaired glucagon response to insulin-induced hypoglycemia during T1DM (Figure 1).

### Myocardial Infarction-Related Inflammation

Myocardial infarction (MI) is one of the most important causes of morbidity and mortality worldwide, with an annual incidence in the United States of 525,000 and 210,000 first and recurrent attacks, respectively. An estimated number of 155,000 silent attacks also occur annually (135). MI causes sterile inflammation of the myocardium characterized by the recruitment of innate and adaptive IS cells (136, 137). In fact, a rapid influx of neutrophils and monocytes-derived macrophages has been demonstrated, followed by DCs, T cells, B cells, NK, and NKT cells. The resolutive phase of the inflammatory process culminates with apoptosis of immune cells and reparative fibrosis of the necrotic myocardium.

Interestingly, it has been observed that MI causes two distinct types of myocardial sympathetic denervation: a permanent denervation of the infarct area, because of tissue ischemic necrosis, and a transient denervation of viable peri-infarct myocardium (138). This transient sympathetic denervation of the non-infarct myocardium, apical to the infarct, was demonstrated in dogs, rodents and even in humans, through imaging techniques consisting of the use of radiolabeled compounds (139–141). On the other hand, it has also been observed an up-regulation of NGF and BDNF in the infarct myocardium and its viable border zone, respectively (142). The role of p75NTR in this cardiac sympathetic transient denervation after ischemia has been demonstrated in mice (143). Three days after MI, it was observed a significant sympathetic denervation in the proximal peri-infarct region in WT mice but not in p75NTR-/- counterparts. Since the loss of sympathetic nerve fibers adjacent to the infarct required p75NTR, it was suggested that ischemia induced the expression of a p75NTR ligand mediating axon degeneration outside of the infarct. In addition, BDNF immunostaining was shown on immune cells, within the infarct area (Figure 1).

As it is widely known, sympathetic innervation increases heart rate as well as atrial and ventricle contractility, through β_1_-adrenergic stimulation (7). Some clinical studies have indicated that sympathetic denervation following MI is a risk factor for the development of arrhythmias and cardiac arrest in attack survivors (144–146). However, the transient sympathetic denervation of the viable non-infarct myocardium seems to have a minimal impact on the development of electrical complications (138).

### Skin Inflammatory Diseases

Since a common manifestation of cutaneous inflammatory diseases is pruritus, the interest in the investigation of changes in the innervation in these conditions was placed mainly in the sensory fibers and not in the sympathetic ones (147). As such, it was observed an increase of fibers containing gastrin-releasing peptide and calcitonin gene-related peptide (CGRP), both in the dermis and epidermis of patients suffering from atopic dermatitis (AD), as well as in murine models of AD and acute dry skin (148, 149). In these cases, it has also been shown an increased expression of NGF in keratinocytes, as well as a decrease in the expression of semaphorin 3A, which has been shown to inhibit NGF-induced sprouting of sensory nerves (3–6). On the other hand, TNF-α was shown to enhance NGF production in human keratinocytes (150). Likewise, mast cells, and mast cell-derived TNF-α, promoted the elongation of epidermal and dermal PGP 9.5+ nerves and dermal CGRP+ nerves in a mouse model of oxazolone-induced contact hypersensitivity (151).

Only a few studies evaluated the sympathetic innervation and activity in the skin during inflammation. In frozen tissue sections from skin biopsies of patients with AD, it was observed an increased expression of markers of different fibers by immunohistochemistry, except those for sympathetic fibers (neuropeptide Y and tyrosine-β-hydroxylase). A diminished density of fibers immunolabeled for neuropeptide Y was also observed in biopsies from patients with urticaria and systemic lupus erythematosus skin lesions (152). In the same line, another group evaluated the electrophysiological functioning of the ANS in patients with AD (153) and psoriasis (154). In both cases, a dysfunction in local sympathetic responses was observed, probably accounting for the impairment in sweat glands secretion and skin dryness seen in those conditions (Figure 1).

### Colitis

During the 1990s, early degenerative and later regenerative phases of mucosal nerve fibers were reported in the rat acute inflammation model of intestinal infection with the nematode *Nippostrongylus brasiliensis* (155, 156). The type of nerve fibers was not characterized in these studies. A decrease in the NA release from the myenteric plexus of rats infected with *Trichinella spiralis* was also reported in those years (157). More recently, Boissé et al. (158) demonstrated an abnormal sympathetic neural activity and decreased NA release from sympathetic nerve fibers in bowels during experimental inflammatory bowel disease (IBD). On the other hand, in a mouse model of dextran sulfate sodium-induced colitis, an inhibition of N-type voltage-gated calcium channels in prevertebral sympathetic neurons was observed (159). According to this study, this inhibition may explain the decreased NA release observed both in inflamed and uninflamed regions of experimental IBD.

It's worth noting that the enteric nervous system constitutes a very complex network, with autonomic extrinsic innervation and neuronal plexuses present in the very wall of the gastrointestinal organs (myenteric and submucosal plexuses), exhibiting intricate interactions (160). Importantly, authors working in the field agree that intestinal inflammation leads to significant changes in the structure and functionality of nearly all these different nerve fibers (161–164). Since many of these neural plasticity phenomena involve non-sympathetic nerve fibers, discussing these issues is beyond the scope of this work. However, as above-referred, concerning changes in local SNS functioning, this neural plasticity mainly comprises an impaired sympathetic activity. Since sympathetic innervation of the gastrointestinal tract modulates motility, blood flow, and secretion, these authors proposed that this impairment may contribute to symptom generation during IBD and intestinal inflammation in general (Figure 1).

### Arthritis

Innervation changes were also noted in rheumatoid arthritis (RA). Straub's group assessed the presence of sympathetic fibers and sensory nerve fibers by immunohistochemistry in fresh synovial tissue of 52 patients with RA, 59 patients with osteoarthritis (OA) and 26 controls. They observed a significant reduction in sympathetic fibers along with an increased number of sensory nerve fibers in RA patients, compared to OA patients and controls. At the same time, they found an increased semaphorin 3C and BDNF expression by *in situ* hybridization in samples from RA patients, with double immunohistochemistry revealing that these molecules were expressed in macrophages and fibroblasts (165, 166). In the same line, higher plasma levels of BDNF were also observed in RA patients, as compared to controls (167).

This loss of sympathetic fibers in the joints was consistently replicated in rat type II collagen-induced arthritis [collaborative studies between Straub's group with del Rey and Besedovsky; (168–170)]. Since sympathetic stimulation increases vascular tone in articular vessels, regulating vascular permeability, and synovial fluid production (171, 172) its loss does not have major clinical consequences (Figure 1).

### Chagas Disease

Chagas disease is an anthropozoonosis of the American continent, caused by the protozoan *Trypanosoma cruzi*. It affects an estimated 8–10 million people worldwide with 25 million people living in endemic areas of Latin America (173). Current migration flows have also led to an increased incidence in non-endemic countries (174, 175). Among chronically infected patients, 30–40% can develop organ involvement 10–30 years after acute infection. This chronic phase of the disease is characterized by cardiomyopathy, arrhythmias, and megavisceras like megaesophagus and megacolon. It constitutes a disabling condition, responsible for significant morbidity and mortality among relatively young patients since primo-infection which mostly occurs during childhood in endemic areas is generally symptomless. Whereas the loss of autonomic innervation of involved organs (i.e., the gastrointestinal tract and the myocardium) was found to underlie clinical manifestations decades ago (176), very little is known about mechanisms leading to this sympathetic and parasympathetic impairment. Auto-immune phenomena secondary to chronic inflammation have been proposed in this regard, but definitive evidence is lacking (177). To the best of my knowledge, the existence of inflammation-related neural plasticity in affected organs during chronic Chagas disease has not been investigated so far but would be worth testing.

### Sympathetic Plasticity as a Whole: Potential Clinical and Immunological Significance

The clinical and experimental evidence reviewed here points out to a neural plasticity phenomenon in inflamed non-lymphoid tissues with a particular focus on changes involving sympathetic nerve fibers, the branch of the ANS that is thought to be the main regulator of the activity of the IS (8–11). ACh-immunomodulatory action has also been shown (178, 179), depending on the integrity of the SNS pathway, as well as an immunomodulatory action of the sensory fibers through different neuropeptides (180, 181). Most authors reporting innervation changes in SLOs and non-lymphoid tissues during an inflammatory/immunological response interpreted these findings as pathological and non-specific phenomena (1). On the contrary, there are firm reasons to believe they correspond to a specific neural plasticity adaptive mechanism -leading to a decreased sympathetic activity during such a situation-, likely mediated by neurotrophins and semaphorins acting on their receptors.

Regarding the clinical consequences of decreased sympathetic activity during inflammation, it is worth remembering that SNS modulate nearly all physiological functions, i.e., cardiovascular, gastrointestinal, respiratory, endocrine, sexual, and temperature regulation, transmitting signals from the central nervous system (CNS) and favoring the homeostatic adaptation to different situations (7). Then, as commented in the present work, an autonomic dysfunction would become clinically evident in many organs and tissues during inflammation, partially accounting for symptom generation and clinical manifestations. This would be particularly true in situations in which compensatory programs counterbalancing the impact of an autonomic blockade during the inflammatory/immunological response were insufficient or had failed to evolve (Figure 1). As above stated, this may apply to chronic inflammatory diseases, hypersensitivity reactions (maladaptive immune reactions *per se*), or protracted immune responses failing to eradicate pathogens (i.e., prolonged septicemia). On the other hand, compensatory mechanisms against sympathetic blockade may involve the release of catecholamines by the adrenal medulla -as proposed for shock- or perhaps peptides/amines release by cells from the diffuse neuroendocrine system, present in different organs, since these compounds parallel in some cases ANS actions (182). Provided this hypothesis is true, sympathetic dysfunction may be at the basis of pathophysiological processes in different inflammatory conditions, opening new research horizons and future directions for novel therapeutic approaches.

As to the immunological significance of the proposed sympathetic neural plasticity mechanism, it may be addressed to change the way by which the NS modulates the IS in its diverse functional-associated activation states. Then, it is necessary to underscore issues about the influence of SNS on IS, both in SLOs and in non-lymphoid organs, along with evidence indicating how the immune response develops in the absence of sympathetic nerves. Importantly, the immune processes that occur in the peripheral non-lymphoid tissues and the SLOs are diverse. In general, DCs recognize, capture and process a given antigen in peripheral tissues and then mature and migrate to SLOs where priming takes place. Subsequently, activated adaptive immune cells are recruited into tissues where the antigen is expressed. Cells of innate immunity are also recruited to the target tissue to eliminate non-self-antigens (183). The SNS has been shown to influence most of these processes (8–11), modulating the different phases of the immune response. But what happens if the sympathetic nerve fibers are not there? Does this process still develop in the same way?

First, as regards to priming in SLOs in absence of sympathetic fibers, to the best of my knowledge, there are no *ad hoc* experiments. Nevertheless, some evidence concerning this was reported in a study employing superantigens, which usually induce a strong proliferative response followed by clonal deletion of a substantial portion of defined VβT cells, with the remaining cells displaying *in vitro* anergy (184, 185). Del Rey and Besedovsky observed that sympathetic denervation prior to the superantigen staphylococcal enterotoxin B (SEB) challenge resulted in decreased SEB-induced T cell proliferation and IL-2 production while hindering the specific deletion of splenic CD4Vβ8 cells seen in intact animals (186, 187). In the same sense, mice lacking dopamine β-hydroxylase (and hence unable to produce NA or Adr but capable of dopamine production) have normal numbers of blood leukocytes, as well as normal T and B cell development and *in vitro* function. However, when challenged *in vivo* with *Listeria monocytogenes* or *Mycobacterium tuberculosis*, they are more susceptible to infection displaying an impaired T cell function, and Th1 cytokine production (188). Similarly, in a murine tuberculosis model, sympathetic denervation with 6-hydroxydopamine (6-OHDA) at the time of mouse infection led to a three-fold higher pulmonary bacillary load at different time-points post-infection. Treated mice also showed a significant increase in the lung parenchyma affected by pneumonia, along with a significant decrease of pro-inflammatory cytokines IFN-γ, TNF-α, IL-12, and IL-17. The same trend of results was observed when administering α/β adrenergic antagonists from day one of infection (189). Likewise, injection of 6-OHDA into rat lateral ventricles–leading to a significant reduction of brain and splenic catecholamine contents–is known to result in a decreased lymphocyte proliferation from spleen and peripheral blood samples, as well as a reduced splenic IL-2 and IFN-γ production and IL-2 mRNA expression (190). Coincidently, mice injected intrastriatally with 6-OHDA have impaired resistance to *L. monocytogenes* along with a reduced immune response to keyhole limpet hemocyanin (191). Rat studies also revealed an age-associated decline in sympathetic innervation in the SLOs accompanied by a significant reduction in IL-2 and IFN-γ production, and T cell proliferation (192). Moreover, peripheral sympathectomy induced by 6-OHDA has been shown to significantly increase CD4+Foxp3+ Treg compartment within SLOs in mice, inhibiting the induction of experimental autoimmune encephalomyelitis (193, 194).

On the other hand, it is well-known that whereas preganglionic sympathetic neurons lie in the intermediate zone of the thoracolumbar spinal cord, sympathetic premotor neurons, and sympathetic neurons antecedent to them, are located in the brain stem, hypothalamus, and telencephalon. Thus, CNS injury affecting those centers may lead to significant changes in SLOs' sympathetic activity, probably influencing the immune response. Remarkably, patients with CNS damage are known to present a secondary immunodeficiency—CNS injury-induced immunodepression—(195–200), characterized by an impaired T- and NK-cell function, both in the acute and chronic phases of the CNS injury. High levels of circulating catecholamines—most likely from adrenal origin—, along with increased plasma corticosteroids levels are observed in the acute phase of CNS injury, as a part of an acute stress response (195–201). Since the immunosuppressive effect of NA is widely accepted (8–11), most authors linked CNS injury-induced immunodepression to an alleged increased SNS activity. However, direct evidence concerning SNS innervation state and activity in lymphoid organs after CNS injury is lacking; for which such immunosuppressive state may be due instead to a loss of noradrenergic nerve fibers integrity within the spleen. In the same sense, decreased cell-mediated immune functions were related to chronic stress and major depressive disorder (202). In both situations, changes in corticosteroid and catecholamines plasma levels along with splenic histological alterations were found (203, 204), setting the basis for future studies addressing whether such alterations are accompanied by modifications in the splenic noradrenergic fibers activity and structure.

Secondly, concerning the point on how inflammation in non-lymphoid organs develops in the absence of sympathetic innervation, studies in this setting also revealed a reduced recruitment of immune cells to target sites. In patients who developed autoimmune pathologies following a hemiplegia-associated CNS injury, there were unilateral cases of RA (205), scleroderma skin changes (206), psoriatic arthritis (207), and asymmetric rheumatoid vasculitis (208), with unique or predominant involvement of the neurologically non-compromised side. In the same line, a patient developing RA after human immunodeficiency virus-1 infection and hemiplegia experienced a complete clinical remission only in the paralytic limbs (209). Since all these patients with stroke and autoimmune disorders not only presented a deficient motor and sensory innervation but also an autonomic one, it was early thought that these surprising phenomena were due to sympathetic dysfunction. As a matter of fact, in the early ‘50 sympathectomy was used in the treatment of RA with satisfactory results (210). In the same line, patients with stroke showed a significant correlation of side asymmetries between delayed-type hypersensitivity responses and axon reflex vasodilation, a cutaneous test used to assess sympathetic activity (211).

Experimentally, a couple of studies in rat models of RA showed that the prior destruction of the sympathetic innervation by injection of 6-OHDA drastically reduced the joint inflammation compared to untreated controls (212, 213). Nevertheless, it is worth noting that in these models not only joints but also SLOs were sympathetically denervated, for which such reduced joint inflammation may be due to a lack of sympathetic action both on priming and migration of inflammatory cells to joints. More recently, Stangenberg et al. (214) designed an elegant model of experimental arthritis in which a group of mice was unilaterally paralyzed by transecting the sciatic and femoral nerves from one hindlimb. These animals developed asymmetrical arthritis, with highly attenuated inflammation of the denervated paw. To explain results, they studied the transcriptome of endothelial cells (ECs) of denervated hind paws to find that the expression of several genes coding for proteins involved in controlling vascular permeability, rolling, adhesion, and transmigration of immune cells was altered, either negatively or positively. This evidence may be taken to imply that vascular innervation may provide signals to ECs that regulate the transmigration of immune cells. While this denervation-related protective effect could not be associated with a single nerve quality in the above-mentioned work (i.e., with the sympathetic, parasympathetic, or sensory nerves), it is worth reminding that most if not all vessels only possess sympathetic innervation. Hence, the presence of sympathetic fibers seems to be essential for immune cells to infiltrate peripheral tissues.

When addressing the potential immunological significance of decreased noradrenergic activity during an ongoing immune response, Besedovsky and del Rey stated that it represents “*a way of releasing immune cells from the inhibitory effects of NA*” (215). This is in line with the immunosuppressive effect of NA, mainly by acting on β-ARs, the most accepted action of the ANS on immune cells (8–11). Although this may be true, evidence discussed in this section indicates that prior presence of noradrenergic fibers is necessary for the effector immune response to start normally, both in the SLOs as in non-lymphoid peripheral tissues, with increased sympathetic activity at the very onset of such response likely exerting a pro-inflammatory effect (11, 216–219). These apparently opposed actions may depend on a different neurotransmitter concentration, distinct types of receptor stimulation, the existence of cotransmitters acting concomitantly, the timing of neurotransmitters release, the different receptor expression pattern and the type and activation state of the immune cells (11, 220), among other factors probably acting *in vivo*. While discussing these issues is beyond the scope of the present work, provided this neural plasticity program does actually occur once the IS is fully activated, it may also represent a sort of “extrinsic” neural-regulated mechanism of peripheral immune tolerance, encompassing cell-mediated innate and adaptive immune responses, probably attempting to limit their extent and magnitude. Hypothetically, within SLOs where priming occurs, once the IS is already activated against a specific antigen, this mechanism would prevent new antigenic challenges from leading to further and successive immune activations, potentially detrimental. In this way, during an ongoing immune response against a given antigen, the IS would remain in a kind of “relative refractory state” for other antigens. On the other hand, in non-lymphoid organs and tissues during active inflammation, the proposed mechanism would preclude a massive recruitment of immune cells to the target site, once cells needed to clear the antigen are already there, and hence hindering more tissue damage. Under such evolutionary pressure, this neural plasticity mechanism might have evolved.

Additional findings favoring the present hypothesis come from the field of neuroscience. The emergence of the placenta allowed viviparity in most mammals, affording survival advantages, like a more complex fetal development and protection. Nevertheless, since the fetus represents a semi-allogeneic graft expressing paternally inherited alloantigens, the establishment of local mechanisms ensuring tolerance by the maternal innate and adaptive IS are essential. In this way, several overlapping mechanisms protect the fetus from the maternal IS (221–224). As known for several decades, during pregnancy the uterus undergoes an extensive axonal degeneration of sympathetic fibers followed by regeneration after delivery (225). This remarkable neural plasticity process is mediated by a range of molecules produced by the myometrium under estrogen's influence, including neurotrophins and pro-neurotrophins acting on Trk receptors and p75NTR, and proteins of the semaphorins family (226–233). The significance of this phenomenon is presently unknown. However, when considering the complex relationship between the IS and the SNS, and the viviparity need of an immune-privileged uterus, this neural plasticity process may emerge as an additional mechanism of peripheral immune tolerance allowing pregnancy.

Another important issue to consider is the effect that changes in tissue innervation may have on DCs, which link the innate and the adaptive immune responses and migrate from peripheral tissues to SLOs. Several years ago, Maestroni showed that DCs expressed functional ARs, with receptor stimulation being able to affect their migration, cytokine production, Th1 and Th17 polarization capacities, and antigen uptake (234–240). Other groups showed that DCs also express functional dopamine receptors through which dopamine—the precursor of NA in sympathetic terminals—, may also modify DCs-mediated Th2 differentiation, CD4+ T cell activation, and Th17 differentiation (241, 242). Nevertheless, while the SNS exerts a marked influence on multiple DCs functions [for review (243)], the same neurotransmitter can mediate different and apparently opposed effects depending on the receptor interactions. Perhaps *in vitro* experiments and the use of agonists/antagonists of adrenergic or dopaminergic receptors may not faithfully reproduce what happens *in vivo*. On the other hand, sympathetic fibers also release cotransmitters like ATP and neuropeptide Y (244) that probably may affect DCs functions, as suggested for peptidergic nerve fibers (245), and hence relevant considering that sensory innervation seems to be increased in inflamed non-lymphoid tissues (147–149, 151). Taken together, there seems to be no conclusive experimental evidence as to the meaning of an immune response to an antigen presented by DCs coming from a peripheral tissue lacking sympathetic innervation or having other possible changes such as increased sensory innervation. Whatever the case, if the retraction of the sympathetic terminals does really represent an extrinsic mechanism of peripheral immune tolerance, DCs migrating from tissues with reduced sympathetic innervation may convey tolerance.

Although much work is needed to corroborate or not the experimental consequences of this hypothesis, it could have a critical impact on fundamental clinical settings wherein peripheral immune tolerance mechanisms are put into place, like transplantation, cancer, and autoimmune pathology, as well as in mucosal immune tolerance (246–248). Accordingly, it seems crucial to study the sympathetic innervation state in these circumstances, both in SLOs and in non-lymphoid target tissues.

## Author Contributions

The author confirms being the sole contributor of this work and has approved it for publication.

### Conflict of Interest Statement

The author declares that the research was conducted in the absence of any commercial or financial relationships that could be construed as a potential conflict of interest.

## References

[1] Bottasso E Toward the existence of a sympathetic neuroplasticity adaptive mechanism influencing the immune response. A hypothetical view-part I. Front Endocrinol. (2019) 10:632 10.3389/fendo.2019.00632 PMC6763740 31616373

[2] Kemi C Grunewald J Eklund A Höglund CO. Differential regulation of neurotrophin expression in human bronchial smooth muscle cells. Respir Res. (2006) 7:18. 10.1186/1465-9921-7-18 16441896 PMC1386667

[3] Tominaga M Ogawa H Takamori K. Decreased production of semaphorin 3A in the lesional skin of atopic dermatitis. Br J Dermatol. (2008) 158:842–4. 10.1111/j.1365-2133.2007.08410.x 18241279

[4] Tominaga M Ozawa S Ogawa H Takamori K. A hypothetical mechanism of intraepidermal neurite formation in NC/Nga mice with atopic dermatitis. J Dermatol Sci. (2007) 46:199–210. 10.1016/j.jdermsci.2007.02.002 17350228

[5] Tominaga M Ozawa S Tengara S Ogawa H Takamori K. Intraepidermal nerve fibers increase in dry skin of acetone-treated mice. J Dermatol Sci. (2007) 48:103–11. 10.1016/j.jdermsci.2007.06.003 17643268

[6] Kamo A Tominaga M Tengara S Ogawa H Takamori K. Inhibitory effects of UV-based therapy on dry skin-inducible nerve growth in acetone-treated mice. J Dermatol Sci. (2011) 62:91–7. 10.1016/j.jdermsci.2011.01.004 21458246

[7] Jänig W editor. Functional anatomy of the peripheral sympathetic and parasympathetic system. In: The Integrative Action of the Autonomic Nervous System: Neurobiology of Homeostasis. Cambridge, UK: Cambridge University Press (2006). p. 13–34. 10.1017/CBO9780511541667.004

[8] Elenkov IJ Wilder RL Chrousos GP Vizi ES The sympathetic nerve–an integrative interface between two supersystems: the brain and the immune system. Pharmacol Rev. (2000) 52:595–638. 11121511

[9] Padro CJ Sanders VM. Neuroendocrine regulation of inflammation. Semin Immunol. (2014) 26:357–68. 10.1016/j.smim.2014.01.003 24486056 PMC4116469

[10] Kenney MJ Ganta CK. Autonomic nervous system and immune system interactions. Compr Physiol. (2014) 4:1177–200. 10.1002/cphy.c130051 24944034 PMC4374437

[11] Bellinger DL Lorton D. Sympathetic nerve hyperactivity in the spleen: causal for nonpathogenic-driven chronic Immune-Mediated Inflammatory Diseases (IMIDs)? Int J Mol Sci. (2018) 19:E1188. 10.3390/ijms19041188 29652832 PMC5979464

[12] Singer M Deutschman CS Seymour CW Shankar-Hari M Annane D Bauer M The Third International Consensus Definitions for Sepsis and Septic Shock (Sepsis-3). JAMA. (2016) 315:801–10. 10.1001/jama.2016.0287 26903338 PMC4968574

[13] Gotts JE Matthay MA. Sepsis: pathophysiology and clinical management. BMJ. (2016) 353:i1585. 10.1136/bmj.i1585 27217054

[14] Gyawali B Ramakrishna K Dhamoon AS. Sepsis: The evolution in definition, pathophysiology, and management. SAGE Open Med. (2019) 7:2050312119835043. 10.1177/2050312119835043 30915218 PMC6429642

[15] László I Trásy D Molnár Z Fazakas J. Sepsis: from pathophysiology to individualized patient care. J Immunol Res. (2015) 2015:510436. 10.1155/2015/510436 26258150 PMC4518174

[16] Sharawy N. Vasoplegia in septic shock: do we really fight the right enemy? J Crit Care. (2014) 29:83–7. 10.1016/j.jcrc.2013.08.021 24095623

[17] Kakihana Y Ito T Nakahara M Yamaguchi K Yasuda T. Sepsis-induced myocardial dysfunction: pathophysiology and management. J Intensive Care. (2016) 4:22. 10.1186/s40560-016-0148-1 27011791 PMC4804632

[18] Burgdorff AM Bucher M Schumann J. Vasoplegia in patients with sepsis and septic shock: pathways and mechanisms. J Int Med Res. (2018) 46:1303–10. 10.1177/0300060517743836 29332515 PMC6091823

[19] Levy B Collin S Sennoun N Ducrocq N Kimmoun A Asfar P Perez P. Vascular hyporesponsiveness to vasopressors in septic shock: from bench to bedside. Intensive Care Med. (2010) 36:2019–29. 10.1007/s00134-010-2045-8 20862451

[20] Shaefi S Mittel A Klick J Evans A Ivascu NS Gutsche J. Vasoplegia after cardiovascular procedures-pathophysiology and targeted therapy. J Cardiothorac Vasc Anesth. (2018) 32:1013–22. 10.1053/j.jvca.2017.10.032 29223724

[21] Cunnion RE Parrillo JE. Myocardial dysfunction in sepsis. Crit Care Clin. (1989) 5:99–118. 10.1016/S0749-0704(18)30452-4 2647229

[22] Lefer AM Martin J. Origin of myocardial depressant factor in shock. Am J Physiol. (1970) 218:1423–7. 10.1152/ajplegacy.1970.218.5.1423 5438272

[23] Rudiger A Singer M. Mechanisms of sepsis-induced cardiac dysfunction. Crit Care Med. (2007) 35:1599–608. 10.1097/01.CCM.0000266683.64081.02 17452940

[24] Ly NP Komatsuzaki K Fraser IP Tseng AA Prodhan P Moore KJ. Netrin-1 inhibits leukocyte migration *in vitro* and *in vivo*. Proc Natl Acad Sci USA. (2005) 102:14729–34. 10.1073/pnas.0506233102 16203981 PMC1253572

[25] Rosenberger P Schwab JM Mirakaj V Masekowsky E Mager A Morote-Garcia JC. Hypoxia-inducible factor-dependent induction of netrin-1 dampens inflammation caused by hypoxia. Nat Immunol. (2009) 10:195–202. 10.1038/ni.1683 19122655

[26] Borsini A Zunszain PA Thuret S Pariante CM. The role of inflammatory cytokines as key modulators of neurogenesis. Trends Neurosci. (2015) 38:145–57. 10.1016/j.tins.2014.12.006 25579391

[27] Poon VY Choi S Park M. Growth factors in synaptic function. Front Synaptic Neurosci. (2013) 5:6. 10.3389/fnsyn.2013.00006 24065916 PMC3776238

[28] Chernogubova E Cannon B Bengtsson T. Norepinephrine increases glucose transport in brown adipocytes via β3-adrenoceptors through a cAMP, PKA, and PI3-kinase-dependent pathway stimulating conventional and novel PKCs. Endocrinology. (2004) 145:269–80. 10.1210/en.2003-0857 14551227

[29] Cooney GJ Caterson ID Newsholme EA. The effect of insulin and noradrenaline on the uptake of 2-[1-14C]deoxyglucose *in vivo* by brown adipose tissue and other glucose-utilising tissues of the mouse. FEBS Lett. (1985) 188:257–61. 10.1016/0014-5793(85)80383-5 3896847

[30] Shimizu Y Kielar D Minokoshi Y Shimazu T. Noradrenaline increases glucose transport into brown adipocytes in culture by a mechanism different from that of insulin. Biochem J. (1996) 314:485–90. 10.1042/bj3140485 8670061 PMC1217076

[31] Shimizu Y Satoh S Yano H Minokoshi Y Cushman SW Shimazu T. Effects of noradrenaline on the cell-surface glucose transporters in cultured brown adipocytes: novel mechanism for selective activation of GLUT1 glucose transporters. Biochem J. (1998) 330:397–403. 10.1042/bj3300397 9461536 PMC1219153

[32] Inokuma K Ogura-Okamatsu Y Toda C Kimura K Yamashita H Saito M. Uncoupling protein 1 is necessary for norepinephrine-induced glucose utilization in brown adipose tissue. Diabetes. (2005) 54:1385–91. 10.2337/diabetes.54.5.1385 15855324

[33] Abi-Gerges N Tavernier B Mebazaa A Faivre V Paqueron X Payen D. Sequential changes in autonomic regulation of cardiac myocytes after *in vivo* endotoxin injection in rat. Am J Respir Crit Care Med. (1999) 160:1196–204. 10.1164/ajrccm.160.4.9808149 10508807

[34] Zhong J Hwang TC Adams HR Rubin LJ. Reduced L-type calcium current in ventricular myocytes from endotoxemic guinea pigs. Am J Physiol. (1997) 273:H2312–24. 10.1152/ajpheart.1997.273.5.H2312 9374768

[35] Liu S Schreur KD. G protein-mediated suppression of L-type Ca2+ current by interleukin-1 beta in cultured ratventricular myocytes. *Am J Physiol*. (1995) 268:C339–49. Erratum in: Am J Physiol. (1995) 268:section C following table of contents. 10.1152/ajpcell.1995.268.2.C339 7864073

[36] Schmidt H Hoyer D Wilhelm J Söffker G Heinroth K Hottenrott K. The alteration of autonomic function in multiple organ dysfunction syndrome. Crit Care Clin. (2008) 24:149–63. 10.1016/j.ccc.2007.10.003 18241783

[37] Hoyer D Friedrich H Zwiener U Pompe B Baranowski R Werdan K. Prognostic impact of autonomic information in multiple organ dysfunction syndrome patients. Int J Cardiol. (2006) 108:359–69. 10.1016/j.ijcard.2005.05.031 15979171

[38] Godin PJ Buchman TG. Uncoupling of biological oscillators: a complementary hypothesis concerning the pathogenesis of multiple organ dysfunction syndrome. Crit Care Med. (1996) 24:1107–16. 10.1097/00003246-199607000-00008 8674321

[39] Annane D Trabold F Sharshar T Jarrin I Blanc AS Raphael JC. Inappropriate sympathetic activation at onset of septic shock: a spectral analysis approach. Am J Respir Crit Care Med. (1999) 160:458–65. 10.1164/ajrccm.160.2.9810073 10430714

[40] Koyoama S Manning JW Role of sympathetic nerve activity in endotoxin induced hypotension in cats. Cardiovasc. Res. (1985) 19:32–37. 10.1093/cvr/19.1.32 3886138

[41] Sharshar T Gray F Lorin de la Grandmaison G Hopkinson NS Ross E Dorandeu A. Apoptosis of neurons in cardiovascular autonomic centres triggered by inducible nitric oxidesynthase after death from septic shock. Lancet. (2003) 362:1799–805. 10.1016/S0140-6736(03)14899-4 14654318

[42] Corrêa TD Takala J Jakob SM. Angiotensin II in septic shock. Crit Care. (2015) 19:98. 10.1186/s13054-015-0802-3 25886853 PMC4360936

[43] Torpy DJ Chrousos GP. The three-way interactions between the hypothalamic-pituitary-adrenal and gonadal axes and the immune system. Baillieres Clin Rheumatol. (1996) 10:181–98. 10.1016/S1521-6942(06)80039-2 8911646

[44] Silverman MN Pearce BD Biron CA Miller AH. Immune modulation of the hypothalamic-pituitary-adrenal (HPA) axis during viral infection. Viral Immunol. (2005) 18:41–78. 10.1089/vim.2005.18.41 15802953 PMC1224723

[45] Polito A Aboab J Annane D. Adrenal insufficiency in sepsis. Rev Bras Ter Intensiva. (2006) 18:86–94. 10.1590/S0103-507X2006000100014 25310332

[46] Annane D Bellissant E Sebille V Lesieur O Mathieu B Raphael JC. Impaired pressor sensitivity to noradrenaline in septic shock patients with and without impaired adrenal function reserve. Br J Clin Pharmacol. (1998) 46:589–97. 10.1046/j.1365-2125.1998.00833.x 9862249 PMC1873798

[47] Jänig W editor. The peripheral sympathetic and parasympathetic pathways. In: The Integrative Action of the Autonomic Nervous System: Neurobiology of Homeostasis. Cambridge, UK: Cambridge University Press (2006). p. 106–67. 10.1017/CBO9780511541667.007

[48] Kopin IJ Plasma Levels of Catecholamines and Dopamine-β-Hydroxylase. In: Trendelemburg U Weiner N, editors. Catecholamines II. Handbook of Experimental Pharmacology, Vol 90/2. Berlin; Heidelberg: Springer (1989). p. 211–75. 10.1007/978-3-642-73551-6_6

[49] Bocking JK Sibbald WJ Holliday RL Scott S Viidik T. Plasma catecholamine levels and pulmonary dysfunction in sepsis. Surg Gynecol Obstet. (1979) 148:715–9. 432784

[50] Bernardin G Strosberg AD Bernard A Mattei M Marullo S. β-adrenergic receptor-dependent and –independent stimulation of adenylate cyclase is impaired during severe sepsis in humans. Intensive Care Med. (1998) 24:1315–22. 10.1007/s001340050768 9885886

[51] Hahn PY Wang P Tait SM Ba ZF Reich SS Chaudry IH. Sustained elevation in circulating catecholamine levels during polymicrobial sepsis. Shock. (1995) 4:269–73. 10.1097/00024382-199510000-00007 8564555

[52] Hwang TL Lau YT Huang SF Chen MF Liu MS Changes of α_1_-adrenergic receptors in human liver during intraabdominal sepsis. Hepatology. (1994) 20:638–42. 10.1002/hep.1840200314 8076922

[53] Tang C Liu MS. Initial externalization followed by internalization of beta-adrenergic receptors in rat heart during sepsis. Am J Physiol. (1996) 270:254–63. 10.1152/ajpregu.1996.270.1.R254 8769809

[54] Shepherd RE Lang CH McDonough KH. Myocardial adrenergic responsiveness after lethal and nonlethal doses of endotoxin. Am J Physiol. (1987) 252:H410–6. 10.1152/ajpheart.1987.252.2.H410 3028179

[55] Reber LL Hernandez JD Galli SJ. The pathophysiology of anaphylaxis. J Allergy Clin Immunol. (2017) 140:335–348. 10.1016/j.jaci.2017.06.003 28780941 PMC5657389

[56] Kemp SF Lockey RF. Anaphylaxis: a review of causes and mechanisms. J Allergy Clin Immunol. (2002) 110:341–8. 10.1067/mai.2002.126811 12209078

[57] Campbell RL Li JT Nicklas RA Sadosty AT; Members of the Joint Task Force; Practice Parameter Workgroup. Emergency department diagnosis and treatment of anaphylaxis: a practice parameter. Ann Allergy Asthma Immunol. (2014) 113:599–608. 10.1016/j.anai.2014.10.007 25466802

[58] Ring J Beyer K Biedermann T Bircher A Duda D Fischer J. Guideline for acute therapy and management of anaphylaxis: S2 Guideline of the German Society for Allergology and Clinical Immunology (DGAKI), the Association of German Allergologists (AeDA), the Society of Pediatric Allergy and Environmental Medicine (GPA), the German Academy of Allergology and Environmental Medicine (DAAU), the German Professional Association of Pediatricians (BVKJ), the Austrian Society for Allergology and Immunology (ÖGAI), the Swiss Society for Allergy and Immunology (SGAI), the German Society of Anaesthesiology and Intensive Care Medicine (DGAI), the German Society of Pharmacology (DGP), the German Society for Psychosomatic Medicine (DGPM), the German Working Group of Anaphylaxis Training and Education (AGATE) and the patient organization German Allergy and Asthma Association (DAAB). Allergo J Int. (2014) 23:96–112. 10.1007/s40629-014-0009-1 26120521 PMC4479483

[59] Leon A Buriani A Dal Toso R Fabris M Romanello S Aloe L. Mast cells synthesize, store, and release nerve growth factor. Proc Natl Acad Sci USA. (1994) 91:3739–43. 10.1073/pnas.91.9.3739 8170980 PMC43657

[60] Casha S Christie S. A systematic review of intensive cardiopulmonary management after spinal cord injury. J Neurotrauma. (2011) 28:1479–95. 10.1089/neu.2009.1156 20030558 PMC3143388

[61] Popa C Popa F Grigorean VT Onose G Sandu AM Popescu M. Vascular dysfunctions following spinal cord injury. J Med Life. (2010) 3:275–85. 20945818 PMC3019008

[62] Partida E Mironets E Hou S Tom VJ. Cardiovascular dysfunction following spinal cord injury. Neural Regen Res. (2016) 11:189–94. 10.4103/1673-5374.177707 27073353 PMC4810964

[63] Mitchell RN Schoen FJ Blood Vessels. In: Kumar V Abbas AK Aster JC editors. Robbins Basic Pathology. Philadelphia, PA: Elsevier Saunders (2018). p. 361–98.

[64] Golledge J. Abdominal aortic aneurysm: update on pathogenesis and medical treatments. Nat Rev Cardiol. (2019) 16:225–42. 10.1038/s41569-018-0114-9 30443031

[65] Nordon IM Hinchliffe RJ Loftus IM Thompson MM. Pathophysiology and epidemiology of abdominal aortic aneurysms. Nat Rev Cardiol. (2011) 8:92–102. 10.1038/nrcardio.2010.180 21079638

[66] Li H Bai S Ao Q Wang X Tian X Li X. Modulation of immune-inflammatory responses in abdominal aortic aneurysm: emerging molecular targets. J Immunol Res. (2018) 2018:7213760. 10.1155/2018/7213760 29967801 PMC6008668

[67] Galkina E Ley K. Immune and inflammatory mechanisms of atherosclerosis (^*^). Annu Rev Immunol. (2009) 27:165–97. 10.1146/annurev.immunol.021908.132620 19302038 PMC2734407

[68] Libby P. Inflammation in atherosclerosis. Arterioscler Thromb Vasc Biol. (2012) 32:2045–51. 10.1161/ATVBAHA.108.179705 22895665 PMC3422754

[69] Fredman G Tabas I. Boosting inflammation resolution in atherosclerosis: the next frontier for therapy. Am J Pathol. (2017) 187:1211–21. 10.1016/j.ajpath.2017.01.018 28527709 PMC5455064

[70] Hellenthal FA Geenen IL Teijink JA Heeneman S Schurink GW Histological features of human abdominal aortic aneurysm are not related to clinical characteristics. Cardiovasc Pathol. (2009) 18:286–93. 10.1016/j.carpath.2008.06.014 18706832

[71] Rodella LF Rezzani R Bonomini F Peroni M Cocchi MA Hirtler L. Abdominal aortic aneurysm and histological, clinical, radiological correlation. Acta Histochem. (2016) 118:256–62. 10.1016/j.acthis.2016.01.007 26858185

[72] Paulo N Cascarejo J Vouga L. Syphilitic aneurysm of the ascending aorta. Interact Cardiovasc Thorac Surg. (2012) 14:223–5. 10.1093/icvts/ivr067 22159251 PMC3279976

[73] Roberts WC Barbin CM Weissenborn MR Ko JM Henry AC. Syphilis as a cause of thoracic aortic aneurysm. Am J Cardiol. (2015) 116:1298–303. 10.1016/j.amjcard.2015.07.030 26307174

[74] Brown SL Busuttil RW Baker JD Machleder HI Moore WS Barker WF. Bacteriologic and surgical determinants of survival in patients with mycotic aneurysms. J Vasc Surg. (1984) 1:541–7. 10.1016/0741-5214(84)90040-5 6436514

[75] Lee WK Mossop PJ Little AF Fitt GJ Vrazas JI Hoang JK. Infected (mycotic) aneurysms: spectrum of imaging appearances and management. Radiographics. (2008) 28:1853–68. 10.1148/rg.287085054 19001644

[76] Bevan RD. Effect of sympathetic denervation on smooth muscle cell proliferation in the growing rabbit ear artery. Circ Res. (1975) 37:14–9. 10.1161/01.RES.37.1.14 1149183

[77] Chamley JH Campbell GR. Trophic influences of sympathetic nerves and cyclic AMP on differentiation and proliferation of isolated smooth muscle cells in culture. Cell Tissue Res. (1975) 161:497–510. 10.1007/BF00224140 169997

[78] Fronek K. Trophic effect of the sympathetic nervous system on vascular smooth muscle. Ann Biomed Eng. (1983) 11:607–15. 10.1007/BF02364090 6095704

[79] Erlinge D Yoo H Edvinsson L Reis DJ Wahlestedt C. Mitogenic effects of ATP on vascular smooth muscle cells vs. other growth factors and sympathetic cotransmitters. Am J Physiol. (1993) 265:H1089–97. 10.1152/ajpheart.1993.265.4.H1089 7694483

[80] Erlinge D Brunkwall J Edvinsson L. Neuropeptide Y stimulates proliferation of human vascular smooth muscle cells: cooperation with noradrenaline and ATP. Regul Pept. (1994) 50:259–65. 10.1016/0167-0115(94)90006-X 8016410

[81] Zhang H Faber JE. Trophic effect of norepinephrine on arterial intima-media and adventitia is augmented by injury and mediated by different alpha1-adrenoceptor subtypes. Circ Res. (2001) 89:815–22. 10.1161/hh2101.098379 11679412

[82] Dechant G Barde YA. The neurotrophin receptor p75(NTR): novel functions and implications for diseases of the nervous system. Nat Neurosci. (2002) 5:1131–6. 10.1038/nn1102-1131 12404007

[83] Kenchappa RS Tep C Korade Z Urra S Bronfman FC Yoon SO. p75 neurotrophin receptor-mediated apoptosis in sympathetic neurons involves a biphasic activation of JNK and up-regulation of tumor necrosis factor-alpha-converting enzyme/ADAM17. J Biol Chem. (2010) 285:20358–68. 10.1074/jbc.M109.082834 20421303 PMC2888447

[84] Kraemer BR Snow JP Vollbrecht P Pathak A Valentine WM Deutch AY. A role for the p75 neurotrophin receptor in axonal degeneration and apoptosis induced by oxidative stress. J Biol Chem. (2014) 289:21205–16. 10.1074/jbc.M114.563403 24939843 PMC4118083

[85] Bush A. Pathophysiological mechanisms of asthma. Front Pediatr. (2019) 7:68. 10.3389/fped.2019.00068 30941334 PMC6434661

[86] Olin JT Wechsler ME. Asthma: pathogenesis and novel drugs for treatment. BMJ. (2014) 349:g5517. 10.1136/bmj.g5517 25420994

[87] McCracken JL Veeranki SP Ameredes BT Calhoun WJ. Diagnosis and management of asthma in adults: a review. JAMA. (2017) 318:279–90. 10.1001/jama.2017.8372 28719697

[88] Schatz M Rosenwasser L. The allergic asthma phenotype. J Allergy Clin Immunol Pract. (2014) 2:645–8; quiz 649. 10.1016/j.jaip.2014.09.004 25439351

[89] Peters SP. Asthma phenotypes: nonallergic (intrinsic) asthma. J Allergy Clin Immunol Pract. (2014) 2:650–2. 10.1016/j.jaip.2014.09.006 25439352

[90] Szentivanyi A The beta-adrenergic theory of the atopic abnormality in bronchial asthma. J Allergy Clin Immunol. (1968) 42:203–32. 10.1016/S0021-8707(68)90117-2

[91] Lemanske RF Jr Kaliner MA. Autonomic nervous system abnormalities and asthma. Am Rev Respir Dis. (1990) 141:S157–61. 10.1164/ajrccm/141.3_Pt_2.S157 2155565

[92] Jartti T. Asthma, asthma medication and autonomic nervous system dysfunction. Clin Physiol. (2001) 21:260–9. 10.1046/j.1365-2281.2001.00323.x 11318835

[93] Mazzone SB Undem BJ. Vagal afferent innervation of the airways in health and disease. Physiol Rev. (2016) 96:975–1024. 10.1152/physrev.00039.2015 27279650 PMC4982036

[94] Undem BJ Carr MJ. The role of nerves in asthma. Curr Allergy Asthma Rep. (2002) 2:159–65. 10.1007/s11882-002-0011-4 11892096

[95] van der Velden VH Hulsmann AR. Autonomic innervation of human airways: structure, function, and pathophysiology in asthma. Neuroimmunomodulation. (1999) 6:145–59. 10.1159/000026376 10213912

[96] de Jongste JC Jongejan RC Kerrebijn KF. Control of airway caliber by autonomic nerves in asthma and in chronic obstructive pulmonary disease. Am Rev Respir Dis. (1991) 143:1421–6. 10.1164/ajrccm/143.6.1421 2048831

[97] Grundstrom N Andersson ROO. Inhibition of the cholinergic neurotransmission in human airways via prejunctional alpha-2-adrenoceptors. Acta Physiol Scand. (1985) 125:513–7. 10.1111/j.1748-1716.1985.tb07749.x 2867666

[98] Davis C Kannan MS Sympathetic innervations of human tracheal and bronchial smooth muscle. Respir Physiol. (1987) 68:53–61. 10.1016/0034-5687(87)90076-4 3037665

[99] Morales DR Dreischulte T Lipworth BJ Donnan PT Jackson C Guthrie B. Respiratory effect of beta-blocker eye drops in asthma: population-based study and meta-analysis of clinical trials. Br J Clin Pharmacol. (2016) 82:814–22. 10.1111/bcp.13006 27161880 PMC5338116

[100] Morales DR Jackson C Lipworth BJ Donnan PT Guthrie B. Adverse respiratory effect of acute β-blocker exposure in asthma: a systematic review and meta-analysis of randomized controlled trials. Chest. (2014) 145:779–786. 10.1378/chest.13-1235 24202435

[101] Empey DW Laitinen LA Jacobs L Gold WM Nadel JA. Mechanisms of bronchial hyperreactivity in normal subjects after upper respiratory tract infection. Am Rev Respir Dis. (1976) 113:131–9. 1247226 10.1164/arrd.1976.113.2.131

[102] Laitinen LA Elkin RB Empey DW Jacobs L Mills J Nadel JA. Bronchial hyperresponsiveness in normal subjects during attenuated influenza virus infection. Am Rev Respir Dis. (1991) 143:358–61. 10.1164/ajrccm/143.2.358 1990953

[103] Freymuth F Vabret A Gouarin S Petitjean J Campet M: [Epidemiology of respiratory virus infections] Allerg Immunol. (2001) 33:66–9. 11339056

[104] Pérez-Yarza EG Moreno A Lázaro P Mejías A Ramilo O. The association between respiratory syncytial virus infection and the development of childhood asthma: a systematic review of the literature. Pediatr Infect Dis J. (2007) 26:733–9. 10.1097/INF.0b013e3180618c42 17848887

[105] Fjaerli HO Farstad T Rød G Ufert GK Gulbrandsen P Nakstad B. Acute bronchiolitis in infancy as risk factor for wheezing and reduced pulmonary function by seven years in Akershus County, Norway. BMC Pediatr. (2005) 5:31. 10.1186/1471-2431-5-31 16109158 PMC1199604

[106] Mirza S Clay RD Koslow MA Scanlon PD. COPD Guidelines: a review of the 2018 GOLD Report. Mayo Clin Proc. (2018) 93:1488–502. 10.1016/j.mayocp.2018.05.026 30286833

[107] Rossi A Butorac-Petanjek B Chilosi M Cosío BG Flezar M Koulouris N. Chronic obstructive pulmonary disease with mild airflow limitation: current knowledge and proposal for future research - a consensus document from six scientific societies. Int J Chron Obstruct Pulmon Dis. (2017) 12:2593–610. 10.2147/COPD.S132236 28919728 PMC5587130

[108] GBD 2015 Chronic Respiratory Disease Collaborators Global, regional, and national deaths, prevalence, disability-adjusted life years, and years lived with disability for chronic obstructive pulmonary disease and asthma, 1990-2015: a systematic analysis for the Global Burden of Disease Study 2015. Lancet Respir Med. (2017) 5:691–706. 10.1016/S2213-2600(17)30293-X 28822787 PMC5573769

[109] Papaiwannou A Zarogoulidis P Porpodis K Spyratos D Kioumis I Pitsiou G Asthma-chronic obstructive pulmonary disease overlap syndrome (ACOS): current literature review. J Thorac Dis. (2014) 6 Suppl 1:S146–51. 10.3978/j.issn.2072-1439.2014.03.04 PMC3966158 24672688

[110] Brown PJ Greville HW Finucane KE. Asthma and irreversible airflow obstruction. Thorax. (1984) 39:131–6. 10.1136/thx.39.2.131 6701824 PMC459739

[111] Backman KS Greenberger PA Patterson R. Airways obstruction in patients with long-term asthma consistent with irreversible asthma. Chest. (1997) 112:1234–40. 10.1378/chest.112.5.1234 9367462

[112] Vonk JM Jongepier H Panhuysen CIM Schouten JP Bleecker ER Postma DS. Risk factors associated with the presence of irreversible airflow limitation and reduced transfer coefficient in patients with asthma after 26 years of follow up. Thorax. (2003) 58:322–7. 10.1136/thorax.58.4.322 12668795 PMC1746641

[113] Grzela K Litwiniuk M Zagorska W Grzela T. Airway remodeling in chronic obstructive pulmonary disease and asthma: the role of matrix metalloproteinase-9. Arch Immunol Ther Exp (Warsz). (2016) 64:47–55. 10.1007/s00005-015-0345-y 26123447 PMC4713715

[114] Lambert RK Wiggs BR Kuwano K Hogg JC Paré PD. Functional significance of increased airway smooth muscle in asthma and COPD. J Appl Physiol. (1993) 74:2771–81. 10.1152/jappl.1993.74.6.2771 8365980

[115] Meurs H Dekkers BG Maarsingh H Halayko AJ Zaagsma J Gosens R. Muscarinic receptors on airway mesenchymal cells: novel findings for an ancient target. Pulm Pharmacol Ther. (2013) 26:145–55. 10.1016/j.pupt.2012.07.003 22842340

[116] Kistemaker LE Oenema TA Meurs H Gosens R. Regulation of airway inflammation and remodeling by muscarinic receptors: perspectives on anticholinergic therapy in asthma and COPD. Life Sci. (2012) 91:1126–33. 10.1016/j.lfs.2012.02.021 22406302

[117] Koarai A Ichinose M. Possible involvement of acetylcholine-mediated inflammation in airway diseases. Allergol Int. (2018) 67:460–6. 10.1016/j.alit.2018.02.008 29605098

[118] Gosens R Gross N. The mode of action of anticholinergics in asthma. Eur Respir J. (2018) 52:1701247. 10.1183/13993003.01247-2017 30115613 PMC6340638

[119] DeFronzo RA Ferrannini E Groop L Henry RR Herman WH Holst JJ Type 2 diabetes mellitus. Nat Rev Dis Primers. (2015) 1:15019 10.1038/nrdp.2015.39 27189025

[120] Katsarou A Gudbjörnsdottir S Rawshani A Dabelea D Bonifacio E Anderson BJ. Type 1 diabetes mellitus. Nat Rev Dis Primers. (2017) 3:17016. 10.1038/nrdp.2017.16 28358037

[121] Krogvold L Wiberg A Edwin B Buanes T Jahnsen FL Hanssen KF. Insulitis and characterisation of infiltrating T cells in surgical pancreatic tail resections from patients at onset of type 1 diabetes. Diabetologia. (2016) 59:492–501. 10.1007/s00125-015-3820-4 26602422

[122] Krogvold L Edwin B Buanes T Ludvigsson J Korsgren O Hyöty H. Pancreatic biopsy by minimal tail resection in live adult patients at the onset of type 1 diabetes: experiences from the DiViD study. Diabetologia. (2014) 57:841–3. 10.1007/s00125-013-3155-y 24429579

[123] Imagawa A Hanafusa T Tamura S Moriwaki M Itoh N Yamamoto K. Pancreatic biopsy as a procedure for detecting in situ autoimmune phenomena in type 1 diabetes: close correlation between serological markers and histological evidence of cellular autoimmunity. Diabetes. (2001) 50:1269–73. 10.2337/diabetes.50.6.1269 11375326

[124] Bottazzo GF Dean BM McNally JM MacKay EH Swift PG Gamble DR. In situ characterization of autoimmune phenomena and expression of HLA molecules in the pancreas in diabetic insulitis. N Engl J Med. (1985) 313:353–60. 10.1056/NEJM198508083130604 3159965

[125] Ahrén B Autonomic regulation of islet hormone secretion–implications for health and disease. Diabetologia. (2000) 43:393–410. 10.1007/s001250051322 10819232

[126] Taborsky GJ Jr. The physiology of glucagon. J Diabetes Sci Technol. (2010) 4:1338–44. 10.1177/193229681000400607 21129328 PMC3005043

[127] Taborsky GJ Jr Mundinger TO. The role of the autonomic nervous system in mediating the glucagon response to hypoglycemia. Endocrinology. (2012) 153:1055–62. 10.1210/en.2011-2040 22315452 PMC3384078

[128] Gerich JE Langlois M Noacco C Karam JH Forsham PH. Lack of glucagon response to hypoglycemia in diabetes: evidence for an intrinsic pancreatic alpha cell defect. Science. (1973) 182:171–3. 10.1126/science.182.4108.171 4581053

[129] Mundinger TO Taborsky GJ Jr. Early sympathetic islet neuropathy in autoimmune diabetes: lessons learned and opportunities for investigation. Diabetologia. (2016) 59:2058–67. 10.1007/s00125-016-4026-0 27342407 PMC6214182

[130] Mei Q Mundinger TO Lernmark A Taborsky GJ Jr. Early, selective, and marked loss of sympathetic nerves from the islets of BioBreeder diabetic rats. *Diabetes*. (2002) 51:2997–3002. Erratum in: Diabetes. (2002) 51:3591. 10.2337/diabetes.51.10.2997 12351439

[131] Taborsky GJ Jr Mei Q Hackney DJ Figlewicz DP LeBoeuf R Mundinger TO. Loss of islet sympathetic nerves and impairment of glucagon secretion in the NOD mouse: relationship to invasive insulitis. Diabetologia. (2009) 52:2602–11. 10.1007/s00125-009-1494-5 19798480

[132] Taborsky GJ Jr Mei Q Bornfeldt KE Hackney DJ Mundinger TO. The p75 neurotrophin receptor is required for the major loss of sympathetic nerves from islets under autoimmune attack. Diabetes. (2014) 63:2369–79. 10.2337/db13-0778 24608438 PMC4066345

[133] Mundinger TO Mei Q Foulis AK Fligner CL Hull RL Taborsky GJ Jr. Human Type 1 diabetes is characterized by an early, marked, sustained, and islet-selective loss of sympathetic nerves. Diabetes. (2016) 65:2322–30. 10.2337/db16-0284 27207540 PMC4955989

[134] Taborsky GJ Jr Mei Q Hackney DJ Mundinger TO. The search for the mechanism of early sympathetic islet neuropathy in autoimmune diabetes. Diabetes Obes Metab. (2014) 16(Suppl. 1):96–101. 10.1111/dom.12341 25200302 PMC4159727

[135] Mozaffarian D Benjamin EJ Go AS Arnett DK Blaha MJ Cushman M. Heart disease and stroke statistics−2015 update: a report from the American Heart Association. Circulation. (2015) 131:e29–322. 10.1161/CIR.0000000000000152 25520374

[136] Francis Stuart SD De Jesus NM Lindsey ML Ripplinger CM. The crossroads of inflammation, fibrosis, and arrhythmia following myocardial infarction. J Mol Cell Cardiol. (2016) 91:114–22. 10.1016/j.yjmcc.2015.12.024 26739214 PMC4764395

[137] Ong SB Hernández-Reséndiz S Crespo-Avilan GE Mukhametshina RT Kwek XY Cabrera-Fuentes HA. Inflammation following acute myocardial infarction: Multiple players, dynamic roles, and novel therapeutic opportunities. Pharmacol Ther. (2018) 186:73–87. 10.1016/j.pharmthera.2018.01.001 29330085 PMC5981007

[138] Parrish DC Francis Stuart SD Olivas A Wang L Nykjaer A Ripplinger CM Transient denervation of viable myocardium after myocardial infarction does not alter arrhythmia susceptibility. Am J Physiol Heart Circ Physiol. (2018) 314:H415–23. 10.1152/ajpheart.00300.2017 29101167 PMC5899257

[139] Stanton MS Tuli MM Radtke NL Heger JJ Miles WM Mock BH. Regional sympathetic denervation after myocardial infarction in humans detected noninvasively using I-123-metaiodobenzylguanidine. J Am Coll Cardiol. (1989) 14:1519–26. 10.1016/0735-1097(89)90391-4 2809013

[140] Kammerling JJ Green FJ Watanabe AM Inoue H Barber MJ Henry DP. Denervation supersensitivity of refractoriness in noninfarcted areas apical to transmural myocardial infarction. Circulation. (1987) 76:383–93. 10.1161/01.CIR.76.2.383 3038369

[141] Li W Knowlton D Van Winkle DM Habecker BA. Infarction alters both the distribution and noradrenergic properties of cardiac sympathetic neurons. Am J Physiol Heart Circ Physiol. (2004) 286:H2229–36. 10.1152/ajpheart.00768.2003 14726300

[142] Hiltunen JO Laurikainen A Väkevä A Meri S Saarma M. Nerve growth factor and brain-derived neurotrophic factor mRNAs are regulated in distinct cell populations of rat heart after ischaemia and reperfusion. J Pathol. (2001) 194:247–53. 10.1002/path.878 11400155

[143] Lorentz CU Parrish DC Alston EN Pellegrino MJ Woodward WR Hempstead BL. Sympathetic denervation of peri-infarct myocardium requires the p75 neurotrophin receptor. Exp Neurol. (2013) 249:111–9. 10.1016/j.expneurol.2013.08.015 24013014 PMC3826885

[144] Boogers MJ Borleffs CJ Henneman MM van Bommel RJ van Ramshorst J Boersma E. Cardiac sympathetic denervation assessed with 123-iodine metaiodobenzylguanidine imaging predicts ventricular arrhythmias in implantable cardioverter-defibrillator patients. J Am Coll Cardiol. (2010) 55:2769–77. 10.1016/j.jacc.2009.12.066 20538172

[145] Fallavollita JA Heavey BM Luisi AJ Jr Michalek SM Baldwa S Mashtare TL Jr. Regional myocardial sympathetic denervation predicts the risk of sudden cardiac arrest in ischemic cardiomyopathy. J Am Coll Cardiol. (2014) 63:141–9. 10.1016/j.jacc.2013.07.096 24076296 PMC3954563

[146] Nishisato K Hashimoto A Nakata T Doi T Yamamoto H Nagahara D. Impaired cardiac sympathetic innervation and myocardial perfusion are related to lethal arrhythmia: quantification of cardiac tracers in patients with ICDs. J Nucl Med. (2010) 51:1241–9. 10.2967/jnumed.110.074971 20679471

[147] Tominaga M Takamori K Recent advances in pathophysiological mechanisms of itch. Expert Rev Dermatol. (2010) 5:197–212. 10.1586/edm.10.7

[148] Tominaga M Takamori K. Itch and nerve fibers with special reference to atopic dermatitis: therapeutic implications. J Dermatol. (2014) 41:205–12. 10.1111/1346-8138.12317 24628070

[149] Tominaga M Ogawa H Takamori K. Histological characterization of cutaneous nerve fibers containing gastrin-releasing peptide in NC/Nga mice: an atopic dermatitis model. J Invest Dermatol. (2009) 129:2901–5. 10.1038/jid.2009.188 19571818

[150] Takaoka K Shirai Y Saito N. Inflammatory cytokine tumor necrosis factor-alpha enhances nerve growth factor production in human keratinocytes, HaCaT cells. J Pharmacol Sci. (2009) 111:381–91. 10.1254/jphs.09143FP 19942804

[151] Kakurai M Monteforte R Suto H Tsai M Nakae S Galli SJ. Mast cell-derived tumor necrosis factor can promote nerve fiber elongation in the skin during contact hypersensitivity in mice. Am J Pathol. (2006) 169:1713–21. 10.2353/ajpath.2006.060602 17071594 PMC1780201

[152] Tobin D Nabarro G Baart de la Faille H van Vloten WA van der Putte SC Schuurman HJ. Increased number of immunoreactive nerve fibers in atopic dermatitis. J Allergy Clin Immunol. (1992) 90:613–22. 10.1016/0091-6749(92)90134-N 1383306

[153] Cicek D Kandi B Berilgen MS Bulut S Tekatas A Dertlioglu SB. Does autonomic dysfunction play a role in atopic dermatitis? Br J Dermatol. (2008) 159:834–8. 10.1111/j.1365-2133.2008.08756.x 18652587

[154] Haligür BD Cicek D Bulut S Berilgen MS. The investigation of autonomic functions in patients with psoriasis. Int J Dermatol. (2012) 51:557–63. 10.1111/j.1365-4632.2011.05111.x 22515580

[155] Stead RH Kosecka-Janiszewska U Oestreicher AB Dixon MF Bienenstock J. Remodeling of B-50 (GAP-43)- and NSE-immunoreactive mucosal nerves in the intestines of rats infected with Nippostrongylus brasiliensis. J Neurosci. (1991) 11:3809–21. 10.1523/JNEUROSCI.11-12-03809.1991 1836018 PMC6575276

[156] Stead RH. Nerve remodelling during intestinal inflammation. Ann N Y Acad Sci. (1992) 664:443–55. 10.1111/j.1749-6632.1992.tb39782.x 1456667

[157] Swain MG Blennerhassett PA Collins SM. Impaired sympathetic nerve function in the inflamed rat intestine. Gastroenterology. (1991) 100:675–82. 10.1016/0016-5085(91)80011-W 1847118

[158] Boissé L Chisholm SP Lukewich MK Lomax AE. Clinical and experimental evidence of sympathetic neural dysfunction during inflammatory bowel disease. Clin Exp Pharmacol Physiol. (2009) 36:1026–33. 10.1111/j.1440-1681.2009.05242.x 19566829

[159] Motagally MA Neshat S Lomax AE. Inhibition of sympathetic N-type voltage-gated Ca2+ current underlies the reduction in norepinephrine release during colitis. Am J Physiol Gastrointest Liver Physiol. (2009) 296:G1077–84. 10.1152/ajpgi.00006.2009 19264956

[160] Jänig W editor. The enteric nervous system. In: The Integrative Action of the Autonomic Nervous System: Neurobiology of Homeostasis. Cambridge, UK: Cambridge University Press (2006). p. 168–207. 10.1017/CBO9780511541667

[161] Lomax AE Sharkey KA Furness JB. The participation of the sympathetic innervation of the gastrointestinal tract in disease states. Neurogastroenterol Motil. (2010) 22:7–18. 10.1111/j.1365-2982.2009.01381.x 19686308

[162] Brierley SM Linden DR. Neuroplasticity and dysfunction after gastrointestinal inflammation. Nat Rev Gastroenterol Hepatol. (2014) 11:611–27. 10.1038/nrgastro.2014.103 25001973

[163] Moynes DM Lucas GH Beyak MJ Lomax AE. Effects of inflammation on the innervation of the colon. Toxicol Pathol. (2014) 42:111–7. 10.1177/0192623313505929 24159054

[164] Lomax AE Pradhananga S Bertrand PP. Plasticity of neuroeffector transmission during bowel inflammation^1^. Am J Physiol Gastrointest Liver Physiol. (2017) 312:G165–70. 10.1152/ajpgi.00365.2016 28082285

[165] Weidler C Holzer C Harbuz M Hofbauer R Angele P Schölmerich J. Low density of sympathetic nerve fibres and increased density of brain derived neurotrophic factor positive cells in RA synovium. Ann Rheum Dis. (2005) 64:13–20. 10.1136/ard.2003.016154 15608299 PMC1755208

[166] Miller LE Weidler C Falk W Angele P Schaumburger J Schölmerich J. Increased prevalence of semaphorin 3C, a repellent of sympathetic nerve fibers, in the synovial tissue of patients with rheumatoid arthritis. Arthritis Rheum. (2004) 50:1156–63. 10.1002/art.20110 15077297

[167] Petersen LE Baptista TSA Molina JK Motta JG do Prado A Piovesan DM. Cognitive impairment in rheumatoid arthritis: role of lymphocyte subsets, cytokines and neurotrophic factors. Clin Rheumatol. (2018) 37:1171–81. 10.1007/s10067-018-3990-9 29372349

[168] del Rey A Wolff C Wildmann J Randolf A Hahnel A Besedovsky HO. Disrupted brain-immune system-joint communication during experimental arthritis. Arthritis Rheum. (2008) 58:3090–9. 10.1002/art.23869 18821705

[169] del Rey A Wolff C Wildmann J Randolf A Straub RH Besedovsky HO. When immune-neuro-endocrine interactions are disrupted: experimentally induced arthritis as an example. Neuroimmunomodulation. (2010) 17:165–8. 10.1159/000258714 20134193

[170] Wolff C Straub RH Hahnel A Randolf A Wildmann J Besedovsky HO. Mimicking disruption of brain-immune system-joint communication results in collagen type II-induced arthritis in non-susceptible PVG rats. Mol Cell Endocrinol. (2015) 415:56–63. 10.1016/j.mce.2015.08.005 26265448

[171] Levick JR. Microvascular architecture and exchange in synovial joints. Microcirculation. (1995) 2:217–33. 10.3109/10739689509146768 8748946

[172] Ferrell WR Khoshbaten A. Responses of blood vessels in the rabbit knee to electrical stimulation of the joint capsule. J Physiol. (1990) 423:569–78. 10.1113/jphysiol.1990.sp018040 1974924 PMC1189775

[173] Pereira PC Navarro EC. Challenges and perspectives of Chagas disease: a review. J Venom Anim Toxins Incl Trop Dis. (2013) 19:34. 10.1186/1678-9199-19-34 24354455 PMC3898031

[174] Malik LH Singh GD Amsterdam EA. Chagas heart disease: an update. Am J Med. (2015) 128:1251.e7–9. 10.1016/j.amjmed.2015.04.036 26052027

[175] Lidani KCF Andrade FA Bavia L Damasceno FS Beltrame MH Messias-Reason IJ. Chagas disease: from discovery to a worldwide health problem. Front Public Health. (2019) 7:166. 10.3389/fpubh.2019.00166 31312626 PMC6614205

[176] Meneghelli UG. Chagas' disease: a model of denervation in the study of digestive tract motility. Braz J Med Biol Res. (1985) 18:255–64. 3939103

[177] Pérez-Molina JA Molina I Chagas disease. Lancet. (2018) 391:82–94. 10.1016/S0140-6736(17)31612-4 28673423

[178] Chavan SS Tracey KJ. Essential neuroscience in immunology. J Immunol. (2017) 198:3389–97. 10.4049/jimmunol.1601613 28416717 PMC5426063

[179] Pavlov VA Chavan SS Tracey KJ. Molecular and functional neuroscience in immunity. Annu Rev Immunol. (2018) 36:783–812. 10.1146/annurev-immunol-042617-053158 29677475 PMC6057146

[180] McMahon SB La Russa F Bennett DL. Crosstalk between the nociceptive and immune systems in host defence and disease. Nat Rev Neurosci. (2015) 16:389–402. 10.1038/nrn3946 26087680

[181] Pinho-Ribeiro FA Verri WA Jr Chiu IM. Nociceptor sensory neuron-immune interactions in pain and inflammation. Trends Immunol. (2017) 38:5–19. 10.1016/j.it.2016.10.001 27793571 PMC5205568

[182] Pearse AG. The cytochemistry and ultrastructure of polypeptide hormone-producing cells of the APUD series and the embryologic, physiologic and pathologic implications of the concept. J Histochem Cytochem. (1969) 17:303–13. 10.1177/17.5.303 4143745

[183] Abbas AK Lichtman AH Pillai S Cellular and Molecular Immunology. 9th ed. Philadelphia, PA: Saunders Elsevier (2017).

[184] Herman A Kappler JW Marrack P Pullen AM. Superantigens: mechanism of T-cell stimulation and role in immune responses. Annu Rev Immunol. (1991) 9:745–72. 10.1146/annurev.iy.09.040191.003525 1832875

[185] MacDonald HR Lees RK Baschieri S Herrmann T Lussow AR. Peripheral T-cell reactivity to bacterial superantigens in vivo: the response/anergy paradox. Immunol Rev. (1993) 133:105–17. 10.1111/j.1600-065X.1993.tb01512.x 8225363

[186] del Rey A Kabiersch A Petzoldt S Randolf A Besedovsky HO. Sympathetic innervation affects superantigen-induced decrease in CD4V beta 8 cells in the spleen. Ann N Y Acad Sci. (2000) 917:575–81. 10.1111/j.1749-6632.2000.tb05423.x 11268386

[187] del Rey A Kabiersch A Petzoldt S Besedovsky HO. Involvement of noradrenergic nerves in the activation and clonal deletion of T cells stimulated by superantigen *in vivo*. J Neuroimmunol. (2002) 127:44–53. 10.1016/S0165-5728(02)00096-6 12044974

[188] Alaniz RC Thomas SA Perez-Melgosa M Mueller K Farr AG Palmiter RD. Dopamine beta-hydroxylase deficiency impairs cellular immunity. Proc Natl Acad Sci USA. (1999) 96:2274–8. 10.1073/pnas.96.5.2274 10051631 PMC26773

[189] Barrios-Payán J Revuelta A Mata-Espinosa D Marquina-Castillo B Villanueva EB Gutiérrez ME. The contribution of the sympathetic nervous system to the immunopathology of experimental pulmonary tuberculosis. J Neuroimmunol. (2016) 298:98–105. 10.1016/j.jneuroim.2016.07.012 27609282

[190] Pacheco-López G Niemi MB Kou W Bildhäuser A Gross CM Goebel MU. Central catecholamine depletion inhibits peripheral lymphocyte responsiveness in spleen and blood. J Neurochem. (2003) 86:1024–31. 10.1046/j.1471-4159.2003.01914.x 12887699

[191] Filipov NM Cao L Seegal RF Lawrence DA. Compromised peripheral immunity of mice injected intrastriatally with six-hydroxydopamine. J Neuroimmunol. (2002) 132:129–39. 10.1016/S0165-5728(02)00321-1 12417443

[192] ThyagaRajan S Madden KS Teruya B Stevens SY Felten DL Bellinger DL. Age-associated alterations in sympathetic noradrenergic innervation of primary and secondary lymphoid organs in female Fischer 344 rats. J Neuroimmunol. (2011) 233:54–64. 10.1016/j.jneuroim.2010.11.012 21186063 PMC3074019

[193] Wirth T Westendorf AM Bloemker D Wildmann J Engler H Mollerus S. The sympathetic nervous system modulates CD4^+^Foxp3^+^ regulatory T cells via noradrenaline-dependent apoptosis in a murine model of lymphoproliferative disease. Brain Behav Immun. (2014) 38:100–10. 10.1016/j.bbi.2014.01.007 24440144

[194] Bhowmick S Singh A Flavell RA Clark RB O'Rourke J Cone RE. The sympathetic nervous system modulates CD4^+^FoxP3^+^ regulatory T cells via a TGF-β-dependent mechanism. J Leukoc Biol. (2009) 86:1275–83. 10.1189/jlb.0209107 19741161 PMC2780915

[195] Prass K Meisel C Höflich C Braun J Halle E Wolf T. Stroke-induced immunodeficiency promotes spontaneous bacterial infections and is mediated by sympathetic activation reversal by poststroke T helper cell type 1-like immunostimulation. J Exp Med. (2003) 198:725–36. 10.1084/jem.20021098 12939340 PMC2194193

[196] Meisel C Schwab JM Prass K Meisel A Dirnagl U. Central nervous system injury-induced immune deficiency syndrome. Nat Rev Neurosci. (2005) 6:775–86. 10.1038/nrn1765 16163382

[197] Prass K Braun JS Dirnagl U Meisel C Meisel A. Stroke propagates bacterial aspiration to pneumonia in a model of cerebral ischemia. Stroke. (2006) 37:2607–12. 10.1161/01.STR.0000240409.68739.2b 16946159

[198] Walter U Kolbaske S Patejdl R Steinhagen V Abu-Mugheisib M Grossmann A. Insular stroke is associated with acute sympathetic hyperactivation and immunodepression. Eur J Neurol. (2013) 20:153–9. 10.1111/j.1468-1331.2012.03818.x 22834894

[199] Winklewski PJ Radkowski M Demkow U. Cross-talk between the inflammatory response, sympathetic activation and pulmonary infection in the ischemic stroke. J Neuroinflammation. (2014) 11:213. 10.1186/s12974-014-0213-4 25539803 PMC4297381

[200] Allison DJ Ditor DS. Immune dysfunction and chronic inflammation following spinal cord injury. Spinal Cord. (2015) 53:14–8. 10.1038/sc.2014.184 25366531

[201] Tibbs PA Young B Ziegler MG McAllister RG Jr Studies of experimental cervical spinal cord transection. Part II: Plasma norepinephrine levels after acute cervical spinal cord transection. J Neurosurg. (1979) 50:629–32. 10.3171/jns.1979.50.5.0629 430158

[202] Reiche EM Nunes SO Morimoto HK. Stress, depression, the immune system, and cancer. Lancet Oncol. (2004) 5:617–25. 10.1016/S1470-2045(04)01597-9 15465465

[203] Veith RC Lewis N Linares OA Barnes RF Raskind MA Villacres EC. Sympathetic nervous system activity in major depression. Basal and desipramine-induced alterations in plasma norepinephrine kinetics. Arch Gen Psychiatry. (1994) 51:411–22. 10.1001/archpsyc.1994.03950050071008 8179465

[204] Hernandez ME Martinez-Mota L Salinas C Marquez-Velasco R Hernandez-Chan NG Morales-Montor J. Chronic stress induces structural alterations in splenic lymphoid tissue that are associated with changes in corticosterone levels in wistar-kyoto rats. Biomed Res Int. (2013) 2013:868742. 10.1155/2013/868742 23533999 PMC3582072

[205] Thompson M Bywaters EG. Unilateral rheumatoid arthritis following hemiplegia. Ann Rheum Dis. (1962) 21:370–7. 10.1136/ard.21.4.370 13981183 PMC1007306

[206] Sethi S Sequeira W. Sparing effect of hemiplegia on scleroderma. Ann Rheum Dis. (1990) 49:999–1000. 10.1136/ard.49.12.999 2270974 PMC1004296

[207] Veale D Farrell M Fitzgerald O. Mechanism of joint sparing in a patient with unilateral psoriatic arthritis and a longstanding hemiplegia. Br J Rheumatol. (1993) 32:413–6. 10.1093/rheumatology/32.5.413 7684307

[208] Dolan AL. Asymmetric rheumatoid vasculitis in a hemiplegic patient. Ann Rheum Dis. (1995) 54:532. 10.1136/ard.54.6.532 7632105 PMC1009922

[209] Lapadula G Iannone F Zuccaro C Covelli M Grattagliano V Pipitone V. Recovery of erosive rheumatoid arthritis after human immunodeficiency virus-1 infection and hemiplegia. J Rheumatol. (1997) 24:747–51. 9101512

[210] Herfort RA. Extended sympathectomy in the treatment of advanced rheumatoid arthritis; a preliminary report. N Y State J Med. (1956) 56:1292–4. 13309705

[211] Tarkowski E Naver H Wallin BG Blomstrand C Tarkowski A. Lateralization of T-lymphocyte responses in patients with stroke. Effect of sympathetic dysfunction? Stroke. (1995) 26:57–62. 10.1161/01.STR.26.1.57 7839398

[212] Aloe L Tuveri MA Levi-Montalcini R. Studies on carrageenan-induced arthritis in adult rats: presence of nerve growth factor and role of sympathetic innervation. Rheumatol Int. (1992) 12:213–6. 10.1007/BF00302155 1290024

[213] Härle P Möbius D Carr DJ Schölmerich J Straub RH. An opposing time-dependent immune-modulating effect of the sympathetic nervous system conferred by altering the cytokine profile in the local lymph nodes and spleen of mice with type II collagen-induced arthritis. Arthritis Rheum. (2005) 52:1305–13. 10.1002/art.20987 15818682

[214] Stangenberg L Burzyn D Binstadt BA Weissleder R Mahmood U Benoist C. Denervation protects limbs from inflammatory arthritis via an impact on the microvasculature. Proc Natl Acad Sci USA. (2014) 111:11419–24. 10.1073/pnas.1410854111 25049388 PMC4128122

[215] del Rey A Besedovsky HO. Immune-neuro-endocrine reflexes, circuits, and networks: physiologic and evolutionary implications. Front Horm Res. (2017) 48:1–18. 10.1159/000452902 28245448

[216] MacNeil BJ Jansen AH Greenberg AH Nance DM. Activation and selectivity of splenic sympathetic nerve electrical activity response to bacterial endotoxin. Am J Physiol. (1996) 270:R264–70. 10.1152/ajpregu.1996.270.1.R264 8769810

[217] Pardini BJ Jones SB Filkins JP. Cardiac and splenic norepinephrine turnovers in endotoxic rats. Am J Physiol. (1983) 245:H276–83. 10.1152/ajpheart.1983.245.2.H276 6349387

[218] Fuchs BA Campbell KS Munson AE. Norepinephrine and serotonin content of the murine spleen: its relationship to lymphocyte beta-adrenergic receptor density and the humoral immune response *in vivo* and *in vitro*. Cell Immunol. (1988) 117:339–51. 10.1016/0008-8749(88)90123-2 2848630

[219] Kohm AP Tang Y Sanders VM Jones SB. Activation of antigen-specific CD4+ Th2 cells and B cells *in vivo* increases norepinephrine release in the spleen and bone marrow. J Immunol. (2000) 165:725–33. 10.4049/jimmunol.165.2.725 10878345

[220] Kin NW Sanders VM. It takes nerve to tell T and B cells what to do. J Leukoc Biol. (2006) 79:1093–104. 10.1189/jlb.1105625 16531560

[221] Thellin O Heinen E. Pregnancy and the immune system: between tolerance and rejection. Toxicology. (2003) 185:179–84. 10.1016/S0300-483X(02)00607-8 12581692

[222] Koch CA Platt JL. Natural mechanisms for evading graft rejection: the fetus as an allograft. Springer Semin Immunopathol. (2003) 25:95–117. 10.1007/s00281-003-0136-0 12955462

[223] Guleria I Sayegh MH. Maternal acceptance of the fetus: true human tolerance. J Immunol. (2007) 178:3345–51. 10.4049/jimmunol.178.6.3345 17339426

[224] Samstein RM Josefowicz SZ Arvey A Treuting PM Rudensky AY. Extrathymic generation of regulatory T cells in placental mammals mitigates maternal-fetal conflict. Cell. (2012) 150:29–38. 10.1016/j.cell.2012.05.031 22770213 PMC3422629

[225] Owman C. Pregnancy induces degenerative and regenerative changes in the autonomic innervation of the female reproductive tract. Ciba Found Symp. (1981) 83:252–79. 10.1002/9780470720653.ch13 6913487

[226] Varol FG Duchemin AM Neff NH Hadjiconstantinou M. Nerve growth factor (NGF) and NGF mRNA change in rat uterus during pregnancy. Neurosci Lett. (2000) 294:58–62. 10.1016/S0304-3940(00)01533-0 11044586

[227] Zoubina EV Smith PG. Sympathetic hyperinnervation of the uterus in the estrogen receptor alpha knock-out mouse. Neuroscience. (2001) 103:237–44. 10.1016/S0306-4522(00)00549-2 11311804

[228] Krizsan-Agbas D Pedchenko T Hasan W Smith PG. Oestrogen regulates sympathetic neurite outgrowth by modulating brain derived neurotrophic factor synthesis and release by the rodent uterus. Eur J Neurosci. (2003) 18:2760–8. 10.1111/j.1460-9568.2003.03029.x 14656325

[229] Richeri A Bianchimano P Mármol NM Viettro L Cowen T Brauer MM. Plasticity in rat uterine sympathetic nerves: the role of TrkA and p75 nerve growth factor receptors. J Anat. (2005) 207:125–34. 10.1111/j.1469-7580.2005.00435.x 16050899 PMC1571519

[230] Brauer MM. Cellular and molecular mechanisms underlying plasticity in uterine sympathetic nerves. Auton Neurosci. (2008) 140:1–16. 10.1016/j.autneu.2008.02.002 18403274

[231] Latini C Frontini A Morroni M Marzioni D Castellucci M Smith PG. Remodeling of uterine innervation. Cell Tissue Res. (2008) 334:1–6. 10.1007/s00441-008-0657-x 18677514

[232] Brauer MM Smith PG Estrogen and female reproductive tract innervation: cellular and molecular mechanisms of autonomic neuroplasticity. Auton Neurosci. (2015) 187:1–17. 10.1016/j.autneu.2014.11.009 25530517 PMC4412365

[233] Brauer MM. Plasticity in uterine innervation: state of the art. Curr Protein Pept Sci. (2017) 18:108–19. 10.2174/1389203717666160322145411 27001066

[234] Maestroni GJ. Dendritic cell migration controlled by alpha 1b-adrenergic receptors. J Immunol. (2000) 165:6743–7. 10.4049/jimmunol.165.12.6743 11120793

[235] Maestroni GJ Mazzola P. Langerhans cells beta 2-adrenoceptors: role in migration, cytokine production, Th priming and contact hypersensitivity. J Neuroimmunol. (2003) 144:91–9. 10.1016/j.jneuroim.2003.08.039 14597102

[236] Maestroni GJ. Sympathetic nervous system influence on the innate immune response. Ann N Y Acad Sci. (2006) 1069:195–207. 10.1196/annals.1351.017 16855146

[237] Maestroni GJ. Short exposure of maturing, bone marrow-derived dendritic cells to norepinephrine: impact on kinetics of cytokine production and Th development. J Neuroimmunol. (2002) 129:106–14. 10.1016/S0165-5728(02)00188-1 12161026

[238] Maestroni GJ. Adrenergic modulation of dendritic cells function: relevance for the immune homeostasis. Curr Neurovasc Res. (2005) 2:169–73. 10.2174/1567202053586776 16181110

[239] Manni M Granstein RD Maestroni G. β2-Adrenergic agonists bias TLR-2 and NOD2 activated dendritic cells towards inducing an IL-17 immune response. Cytokine. (2011) 55:380–6. 10.1016/j.cyto.2011.05.013 21683614 PMC3148409

[240] Yanagawa Y Matsumoto M Togashi H. Enhanced dendritic cell antigen uptake via α_2_ adrenoceptor-mediated PI3K activation following brief exposure to noradrenaline. J Immunol. (2010) 185:5762–8. 10.4049/jimmunol.1001899 20935206

[241] Nakano K Higashi T Takagi R Hashimoto K Tanaka Y Matsushita S. Dopamine released by dendritic cells polarizes Th2 differentiation. Int Immunol. (2009) 21:645–54. 10.1093/intimm/dxp033 19332443

[242] Prado C Contreras F González H Díaz P Elgueta D Barrientos M. Stimulation of dopamine receptor D5 expressed on dendritic cells potentiates Th17-mediated immunity. J Immunol. (2012) 188:3062–70. 10.4049/jimmunol.1103096 22379034

[243] Takenaka MC Guereschi MG Basso AS. Neuroimmune interactions: dendritic cell modulation by the sympathetic nervous system. Semin Immunopathol. (2017) 39:165–176. 10.1007/s00281-016-0590-0 27800584

[244] Burnstock G. Cotransmission in the autonomic nervous system. Handb Clin Neurol. (2013) 117:23–35. 10.1016/B978-0-444-53491-0.00003-1 24095113

[245] Beresford L Orange O Bell EB Miyan JA. Nerve fibres are required to evoke a contact sensitivity response in mice. Immunology. (2004) 111:118–25. 10.1111/j.1365-2567.2004.01786.x 14678206 PMC1782395

[246] Alonso R Flament H Lemoine S Sedlik C Bottasso E Péguillet I. Induction of anergic or regulatory tumor-specific CD4+ T cells in the tumor-draining lymph node. Nat Commun. (2018) 9:2113. 10.1038/s41467-018-04524-x 29844317 PMC5974295

[247] Mowat AM. Anatomical basis of tolerance and immunity to intestinal antigens. Nat Rev Immunol. (2003) 3:331–41. 10.1038/nri1057 12669023

[248] Holmgren J Czerkinsky C. Mucosal immunity and vaccines. Nat Med. (2005) 11:S45–53. 10.1038/nm1213 15812489

